# Insights into the Natural Defenses of a Coral Reef Fish Against Gill Ectoparasites: Integrated Metabolome and Microbiome Approach

**DOI:** 10.3390/metabo10060227

**Published:** 2020-05-30

**Authors:** Miriam Reverter, Pierre Sasal, Marcelino T. Suzuki, Delphine Raviglione, Nicolas Inguimbert, Alan Pare, Bernard Banaigs, Sébastien N. Voisin, Philippe Bulet, Nathalie Tapissier-Bontemps

**Affiliations:** 1 Institut für Chemie und Biologie des Meeres, Carl von Ossietzky Universität Oldenburg, 26382 Wilhelmshaven, Germany; 2 CRIOBE, USR3278-EPHE/CNRS/UPVD/PSL, University of Perpignan Via Domitia, 52 Avenue Paul Alduy, 66860 Perpignan, France; sasal@univ-perp.fr (P.S.); delphine.raviglione@univ-perp.fr (D.R.); nicolas.inguimbert@univ-perp.fr (N.I.); alan.pare@outlook.fr (A.P.); banaigs@univ-perp.fr (B.B.); 3 Laboratoire d’Excellence ‘CORAIL’, Moorea 98729, French Polynesia; 4 Laboratoire de Biodiversité et Biotechnologies Microbiennes, Sorbonne Université, CNRS, USR3579, Observatoire Océanologique, 66650 Banyuls-sur-mer, France; suzuki@obs-banyuls.fr; 5 Plateforme BioPark d’Archamps, ArchParc, 74160 Archamps, France; sebastien.voisin@biopark-archamps.org (S.N.V.); philippe.bulet@biopark-archamps.org (P.B.); 6 CR UGA, IAB, InsermU1209, CNRS UMR 5309, 38700 La Tronche, France

**Keywords:** fish mucus, host-parasite interactions, butterflyfish, metabolomics, microbiome, multi-omics, *Chaetodon*, peptides

## Abstract

Understanding natural defense mechanisms against parasites can be a valuable tool for the development of innovative therapies. We have previously identified a butterflyfish species (*Chaetodon lunulatus*) that avoids gill monogenean parasites while living amongst closely related parasitized species. The metabolome and microbiome of several sympatric butterflyfish species from the island of Moorea (French Polynesia) were previously described. In this study, we used the previously generated datasets in an attempt to identify metabolites and bacteria potentially involved in parasite defense mechanisms. We investigated the interplay between the gill mucus metabolome and microbiome of the non-susceptible *C. lunulatus* versus sympatric butterflyfish species that were always found parasitized in the Central and Eastern Indo-Pacific. After observing significant differences between the metabolome and bacteria of susceptible versus non-susceptible fish, we obtained the discriminant metabolites and operational taxonomic units (OTUs) using a supervised analysis. Some of the most important discriminant metabolites were identified as peptides, and three new peptides derived from β-subunit hemoglobin from *C. lunulatus* (CLHbβ-1, CLHbβ-2, and CLHbβ-3) were purified, characterized and synthesized to confirm their structures. We also identified specific bacterial families and OTUs typical from low-oxygen habitats in *C. lunulatus* gill mucus. By using a correlation network between the two datasets, we found a *Fusobacteriaceae* strain exclusively present in *C. lunulatus* and highly correlated to the peptides. Finally, we discuss the possible involvement of these peptides and *Fusobacteriaceae* in monogenean avoidance by this fish species.

## 1. Introduction

Parasites are crucial for a well-balanced ecosystem, contributing to its organization by modifying competitive and trophic interactions and driving natural selection [1]. However, disruption of ecosystem equilibrium due to activities that degrade the environment and compromise the hosts (e.g., pollution, climate change, culture intensification) or through the introduction of new species that can introduce new pathogens (enemy release hypothesis), can lead to parasite outbreaks and can result in high host mortalities [2,3]. Increasing the connectivity of systems (e.g., global animal trade) also heightens the risks of emergence and transmission of infectious agents [4,5]. Understanding the factors underpinning host-parasite interactions is thus critical to prevent the emergence of infectious diseases that can result in biodiversity decrease and severe economic losses [5,6]. In this context, a better understanding of natural defense mechanisms can contribute to the development of alternative sustainable therapies both in human and animal medicine [7,8].

Host-parasite interactions have been traditionally studied as an interplay between host defense and parasite evasion mechanisms [9]. However, recent research suggests that host and parasite associated-microorganisms play major roles in host and parasite fitness, highlighting the need to study host-parasite interactions in the light of the microbiota [8,10]. Despite the poor understanding of the role of native host microbiota against eukaryotic parasites is poorly understood, some studies suggest they can deploy multiple strategies to control parasitic infections. For example, Cirimotich et al. found that an *Enterobacter* bacterium from mosquito inhibited the development of malaria parasites by the production of reactive oxygen species (ROS), rendering mosquitoes resistant to infection [11]. Other studies in reptiles and teleostean fish showed that associated bacteria could be a source of antibacterial and antifungal metabolites, but few studies have explored their activities against eukaryote parasites [12,13]. The involvement of microbiota in the modulation of the host innate immune system is well recognized in many animals, including fish, and is considered to influence pathogen control strategies [14]. For example, in a recent study, Sepahi and collaborators [15] found that a bacterium from external mucosal surfaces of rainbow trout was able to regulate the symbiont communities and the production of immunoglobulins, T-cells, and B-cells through the production of sphingolipids.

Mucosal surfaces are the main zones of contact between animals and the environment and thus play a key role in both entry and defense against pathogens [16]. Teleost mucus is mainly composed of mucin glycoproteins, which are continuously produced and shed to limit pathogen contact with epithelial cells and promote their clearance [17]. Mucosal surfaces also contain a wide-array of immune-related molecules (e.g., lysozyme, immunoglobulins, lectins, and antimicrobial peptides) and diverse associated microbial communities that orchestrate to control pathogens [14,15,16,18]. Despite the recent increase in fish mucus research, its role in parasite infection and control remains poorly understood. An increasing body of research suggests some fish mucus cues are used by parasites to detect their hosts [19,20], and mucosal macromolecules such as IgM and the glycoprotein WAP 65-2 have been found to attract and induce attachment of several monogenean parasites in tiger pufferfish (*Takifugu rubripes*) [21,22]. Fish mucus is known to display antimicrobial, antifungal, and antiparasitic activities [23,24], but to date, few antiparasitic molecules have been identified. Antimicrobial peptides (AMPs), which are normally small cationic amphipathic peptides with less than 50 amino acids, are commonly found in fish mucus and fish epithelium [25]. Some peptides isolated from gill epithelium (hemoglobin-derived peptide Hbβ-P1 and piscidin-2 are active against several parasites, but whether these AMPs are also found in fish mucus or whether other AMPs isolated from fish mucus display antiparasitic activities is not clear [26,27,28].

Butterflyfishes (Chaetodontidae), a diverse and emblematic family of coral reef fishes, are naturally parasitized by monogeneans belonging to the family Dactylogyridae [29]. Dactylogyrids are direct cycle flatworms that attach to fish gills by two pairs of hooks [30]. Despite the fact that, all parasites are considered to impose a cost on their hosts, it is extremely difficult to determine the physiological impacts of parasites in the natural environment [31]. As such, the effects of dactylogyrids on butterflyfish communities have not been determined, but other *Dactylogyrus* species are recognized as major pathogens in cultured fish [32,33]. In a recent study, we found that only one butterflyfish species (*Chaetodon lunulatus*) out of 34 species studied through the Indo-Pacific (including the present study site, Moorea in French Polynesia), was never parasitized by gill monogeneans [34]. Butterflyfish phylogeny, ecology, and behavior have been studied extensively [35,36], and *C. lunulatus* does not present any striking difference in lifestyle compared to other sympatric and phylogenetically close butterflyfishes such as *C. ornatissmus* and *C. reticulatus*, which are always found highly parasitized [34]. In this study, we aimed to investigate the mechanisms behind the species-specific resistance of *C. lunulatus* to gill monogeneans. We focused on the study of the gill mucus as the main zone of contact between fish and parasites, and we used an integrative approach to identify potential metabolites and operational taxonomic units (OTUs) that might be involved in either parasite attraction or deterrence. This study is a continuation of our previous research on the characterization of different butterflyfish species gill mucus metabolome and microbiome [37,38], focused on the integration of metabolome and microbiome data in an attempt to elucidate parasite attraction or deterrence mechanisms. The use of this approach allowed us to characterize three previously unknown β-subunit hemoglobin-derived peptides from *C. lunulatus* gill mucus and explore the correlations between *C. lunulatus* overexpressed features and the identified bacteria.

## 2. Results

### 2.1. Metabolomic Analysis of Chaetodon Gill Mucus

The metabolomics dataset used in this article was previously obtained and described in Reverter et al. [37]. Metabolomics analyses were performed on both polar (fraction H_2_O/MeOH) and apolar (fraction MeOH/CH_2_Cl_2_) fractions. Principal coordinate analysis (PCoA) of the apolar fraction did not show a significant difference (ANOSIM, *p* = 0.20) between the susceptible and non-susceptible fish (*C. lunulatus*) to gill monogeneans (Supplementary Figure S1). Therefore, we did not perform further analysis and all the following results, including variable of importance projection (VIP) selection, identification, and correlation with bacterial OTUs were based on the analysis of the polar fraction. 

**Figure 1:**
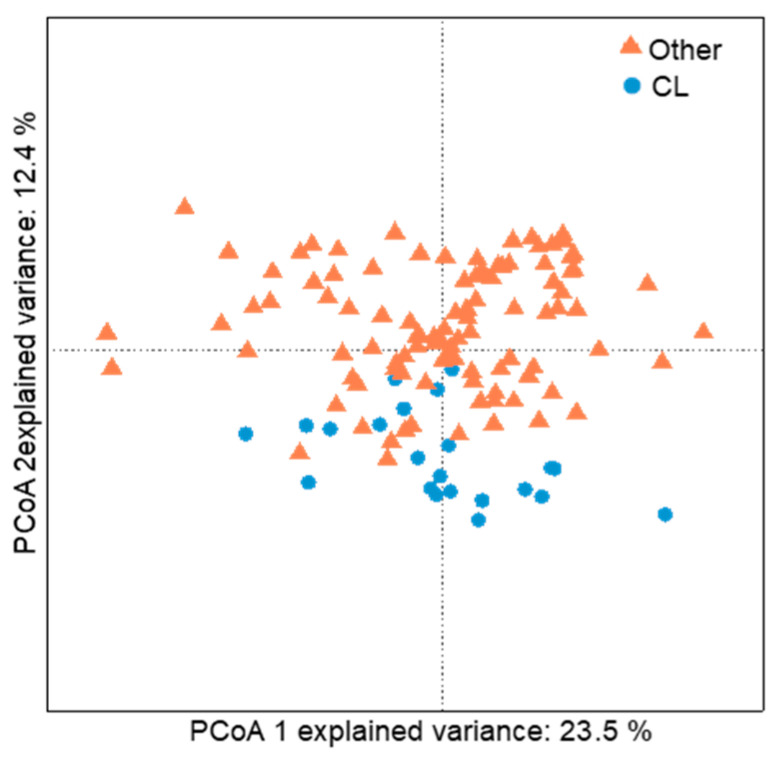
PCoA analyses of gill mucus metabolome of susceptible (other, orange triangles) and non-susceptible (*Chaetodon* *lunulatus*, CL, blue circles) butterflyfish.

**Figure 2:**
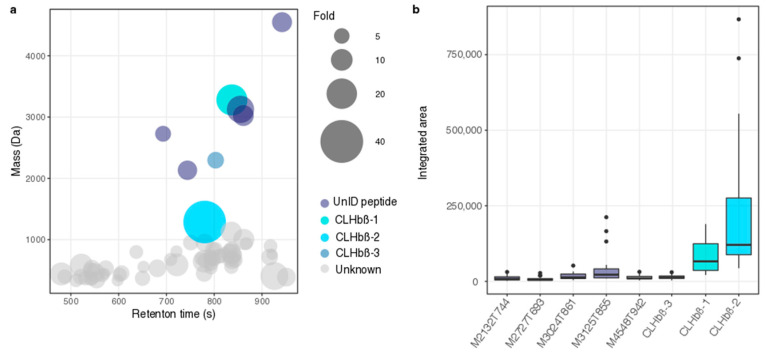
(**a**) Bubble plot displaying the molecular masses in Daltons (Da) and retention time in seconds (s) of significantly over-expressed VIPs in *Chaetodon* *lunulatus*. Bubble size indicates the fold-change between *C. lunulatus* and susceptible butterflyfishes, different colors show different identified (CLHbβ-1, CLHbβ-2, and CLHbβ-3 have been characterized) and unidentified peptides (**b**) Integrated areas of VIP peptides (over-expressed in *C. lunulatus*). For uncharacterized peptides M is their molecular mass (Da) and T their retention time in seconds (s). Median, first, and third quartile are plotted and the dots indicate outliers.

**Figure 3:**
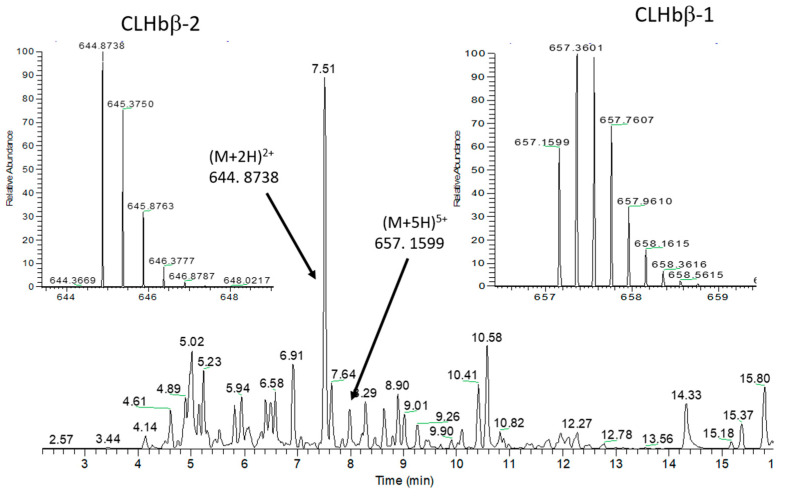
UHPLC-HRMS/MS analysis in positive mode of the peptide enriched fraction of *Chaetodon* *lunulatus* mucus. CLHbβ-1 and CLHbβ-2 are identified in the chromatogram, and their characteristic peptide spectra displaying multicharged ions are shown.

A PCoA of the polar fraction showed significant differences between the gill mucus metabolome composition of susceptible and non-susceptible fish (ANOSIM, *p* < 0.001, Figure 1). The PLS-DA model accurately predicted differences between the metabolic profiles of susceptible and non-susceptible fish (NMC = 0.043, *p*-value = 0.001). After “cleaning” of the matrix (removal of isotopes and adducts), we found 69 features with a VIP score higher than 1, all significantly over-expressed (Kruskal–Wallis, *p* < 0.001) in the non-susceptible fish (Figure 2a). Eight out of the 69 VIPs were identified as peptides due to their characteristic multicharged ions and typical fragmentation patterns observed from the high-resolution mass spectrometry data, whilst the rest of the VIP were unknowns (Figure 2b and Figure 3).

### 2.2. Peptide Characterisation and Synthesis

Since LC-ESI-HRMS/MS profiles of the most important VIPs were characteristic of peptides, we optimized the extraction procedure using an acidic extraction to selectively precipitate large proteins while enhancing the solubility of peptides. After solid-phase extraction (SPE) pre-purification of the acidic extract from *C. lunulatus* mucus, analysis by ultra-high performance liquid chromatography high-resolution tandem mass spectrometry (UHPLC-HRMS/MS) of this sample compared to the polar extract (used for the metabolomics pipeline), showed enrichment in two major peptides with *m*/*z* 657.1599 [M + 5H]^5+^ (CLHbβ-1) and 644.8738 [M + 2H]^2+^ (CLHbβ-2) (Figure 3, Supplementary Figures S2 and S3). Another VIP peptide (CLHbβ-3), not detectable on the Total Ion Chromatogram (TIC) presented in Figure 3, was detected using selected ion extraction mode at *m*/*z* 766.0947 [M + 3H]^3+^ (Supplementary Figure S4).

**Figure 4:**
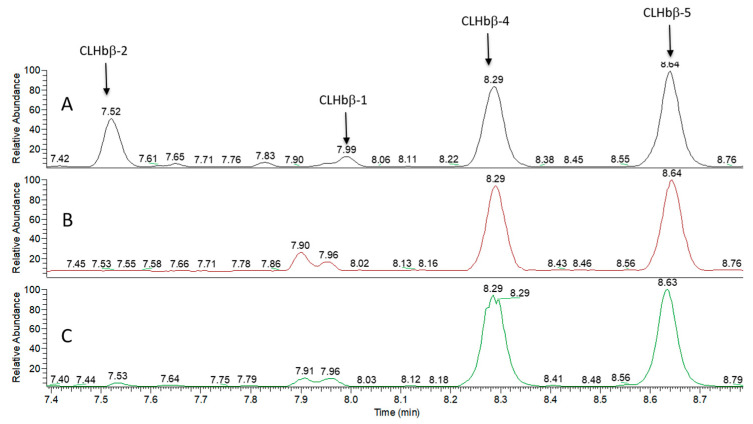
LC-ESI-HRMS analysis of the peptide-enriched fraction of *Chaetodon lunulatus* mucus (**A**), *C. ornatissimus* (**B**) and *C. reticulatus* (**C**) in positive mode, displaying the peptides CLHbβ-1, CLHbβ-2, CLHbβ-4, and CLHbβ-5 (CLHb β-3 was not observable on TIC but was identified in ion extraction mode; see Supplementary Figure S4).

Acidic extractions of two heavily parasitized species (*C. reticulatus* and *C. ornatissimus*) [34] were done as control and did not show any of the VIPs previously observed in the non-susceptible species, even using the selected ion extraction mode. Additionally, the comparison of the TIC obtained from the peptide-enriched fractions extracted from susceptible and non-susceptible fish species highlighted the presence of two additional peptides with *m*/*z* 679.7758 [M + 5H]^5+^ (CLHbβ-4) and 702.3929 [M + 5H]^5+^ (CLHbβ-5) in the mucus samples of all analyzed species (Figure 4, Supplementary Figures S5 and S6). The mass differences between CLHbβ-1 and CLHbβ-4 (113 Da) and CLHbβ-1 versus CLHbβ-5 (226 Da) correspond to one and two additional leucine residues (Figure 4, Table 1).

The SPE pre-purified sample was analyzed for peptide de novo sequencing for confirmation and further characterization of these five peptides. MALDI-MS analysis of the 60% acetonitrile (MeCN) eluted fraction showed the presence of several potential peptides, including five peaks whose MH^+^ *m*/*z* values would match the expected average MH^+^ *m*/*z* values for the five peptides discussed above (Supplementary Figure S7). After nanoLC-MS/MS analysis, de novo sequencing of the spectra of CLHbβ-1, CLHbβ-2 and CLHbβ-3 revealed they were composed of 30, 10, and 22 amino acid residues, respectively (Supplementary Figure S8, Table 1). CLHbβ-4 and CLHbβ-5 were shown to have the same sequence as CLHbβ-1, but with one and two extra leucine on the C-terminal end, respectively.

**Figure 5:**
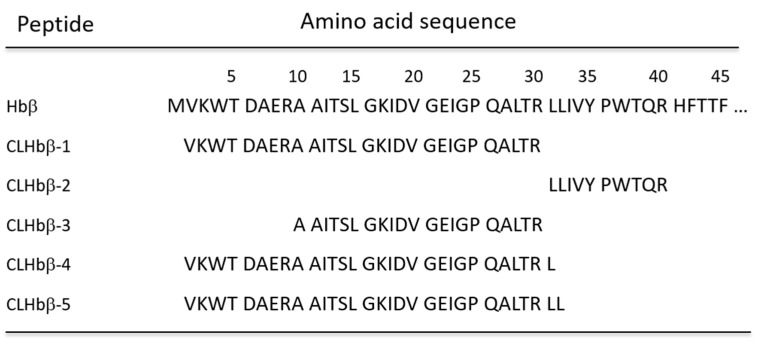
Amino-acid sequences of *Chaetodon austriacus* hemoglobin precursor. Residues 2 to 31, 32 to 41, 10 to 31, 2 to 32, and 2 to 33 correspond to CLHbβ-1, CLHbβ-2, CLHbβ-3, CLHbβ-4, and CLHbβ-5, respectively.

Search for amino acid sequence homology against bioactive peptide databases BIOPEP-UWM (http://www.uwm.edu.pl/biochemia/index.php/pl/biopep) showed no significant sequence identity to any sequence in these databases. Homology search with the Swissprot–UniprotKB database using Mascot and Sequest highlighted peptides derived from the hemoglobin subunit beta-B of the Japanese amberjack *Seriola quinqueradiata* with high scores (see Supplementary Table S1). Further comparison with a beta subunit hemoglobin sequence of a closely related species *Chaetodon austriacus* [39] confirmed that the five VIP peptides showed 100% identity with beta subunit hemoglobin (Hbβ N-terminus (Figure 5).

The five peptides identified (CLHbβ-1 to 5) were synthesized according to Fmoc/tBu solid-phase method using preloaded Leu or Arg Wang resin in order to confirm the previously obtained structures. After elongation was completed, the peptides were cleaved from the resin using TFA, affording targeted compounds with yields ranging from 42% to 92% and a purity of at least 90% for each of the synthetic peptides as judged by mass spectrometry analysis. Peptides CLHbβ-2 to CLHbβ-5 tended to form gels; therefore, they were used without further purification. The accuracy of their sequence was confirmed by comparing the chromatographic (identical retention times) and spectrometric data (i.e., exact mass and fragmentation pattern) between the synthetic and the natural forms (Supplementary Figures S4–S8).

### 2.3. Bacterial Community of Chaetodon Gill Mucus

**Figure 6:**
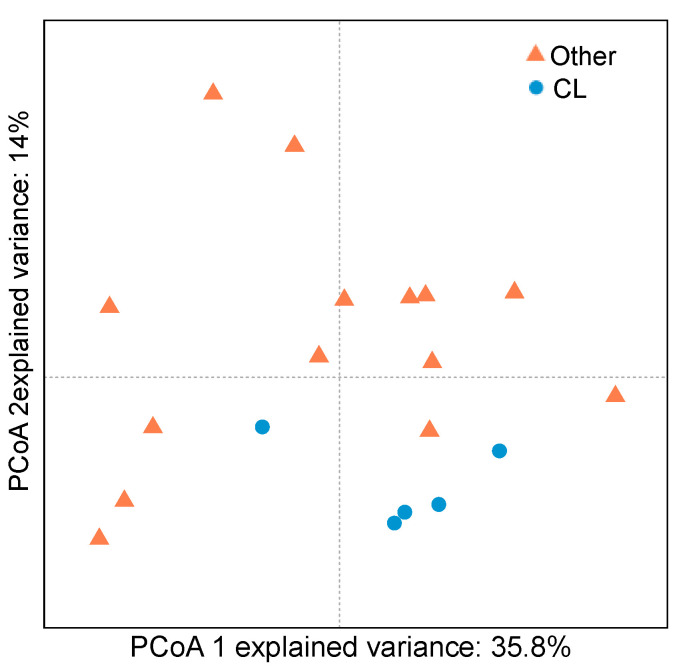
PCoA analyses of gill mucus microbiome of susceptible (other, orange triangles) and non- susceptible (*C. lunulatus*, CL, blue circles) butterflyfish.

**Figure 7:**
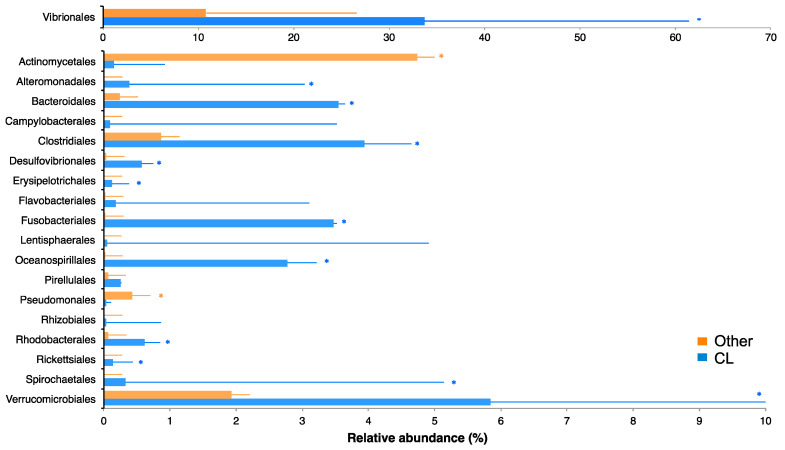
VIP bacterial orders identified between susceptible (orange) and non-susceptible (blue, *C. lunulatus*) fish. * indicates a significant difference (Kruskal–Wallis, *p*-value < 0.05).

The bacterial communities of one non-susceptible butterflyfish (*C. lunulatus*) and three highly susceptible butterflyfish species (*C. ornatissimus, C. reticulatus,* and *C. vagabundus*) have been previously described [38]. Here, we analyzed the same data in the context of parasite presence and absence. PCoA showed significant differences between the gill mucosal bacterial communities of parasitized and non-parasitized fishes (ANOSIM, *p* < 0.001, Figure 6). The PLS-DA model accurately predicted bacterial differences between susceptible and non-susceptible fish (NMC = 0.016, *p*-value = 0.015). We identified 133 OTUs with VIP scores higher than 1, which belonged to 19 bacterial orders (Figure 7). The mean relative abundance of these orders in susceptible and non-susceptible fish was obtained by grouping the 133 retained OTUs by bacterial order. The Actinomycetales and Pseudomonadales were the only bacterial orders that presented a significantly higher mean relative abundance in the susceptible fish (4.7 ± 4.8%, and 0.4 ± 0.5% *p* < 0.005). Twelve bacterial orders presented significantly higher mean relative abundances in non-susceptible fish (Figure 7): Fusobacteriales (3.5 ± 2.9%), Bacteroidales (3.6 ± 2.4%), Oceanospirillales (2.8 ± 4.8%), Alteromonadales (0.4 ± 0.7%), Spirochaetales (0.33 ± 0.30), Rhodobacterales (0.6 ± 0.8%), Desulfovibrionales (0.6 ± 0.6%), Vibrionales (33.7 ± 27.7%), Verrucomicrobiales (5.8 ± 4.8%), Clostridiales (3.9 ± 3.4%), Rickettsiales (0.13 ± 0.2%) and Erysipelotrichales (0.12 ± 0.10%).

### 2.4. Correlation between VIP Metabolites and VIP OTUs

**Figure 8:**
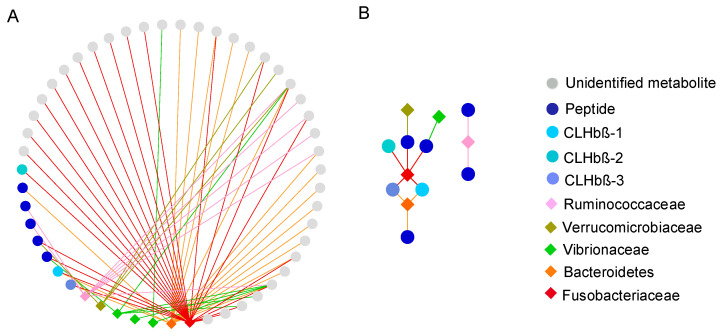
Correlation network (Spearman correlation) between VIP metabolites (circles) and VIP OTUs (diamonds) (**A**) and VIP peptides (circles) and VIP OTUs (diamonds) (**B**). Lines show ρ > 0.5.

A correlation analysis between metabolites and bacterial OTUs that were significantly overexpressed in non-susceptible fish, with a VIP score higher than 1 and present in all *C. lunulatus* individuals, was performed. All peptides were significantly correlated to at least one OTU. We identified seven OTUs that were correlated (ρ ≥ 0.5, *p* < 0.005) to at least one of the VIP metabolites (three Vibrionaceae, one Verrucomicrobiaceae, one Ruminococcaceae, one Fusobacteriaceae and one Bacteroidales that could not be identified to family level). The Fusobacteriaceae and the Bacteroidales were the OTUs correlated to the highest number of metabolites (Figure 8A). The Fusobacteriaceae was also the OTU correlated to the highest number of peptides (4 out of 7) and the only OTU correlated to the three *C. lunulatus* peptides (CLHbβ-1, CLHbβ-2, CLHbβ-3) (Figure 8B). Other OTUs correlated to peptides included the Bacteroidales (correlated to CLHbβ-2 and CLHbβ-3), the Ruminococcaceae, the Verrucomicrobiaceae, and one Vibrionaceae (Figure 8B). 

## 3. Discussion

The emergence of infectious diseases in animal production systems is a major problem causing severe economic losses (estimated US 9.5$ billion per year) through decreased production of farmed species or increased production costs [40]. Treatment of ectoparasite infections in aquaculture normally involves bathing fish in chemical substances (e.g., praziquantel, formaldehyde) that have nefarious environmental consequences, are hazardous for the animals and sometimes display reduced efficacy due to the emergence of resistance [41,42]. Therefore, the development of more environmentally-friendly therapies and sustainable/integrated alternatives is urgently needed [43]. Within this context, a better understanding of the natural defense mechanisms deployed by fish to avoid parasites can be extremely useful for the development of innovative therapies. In this study, we have investigated the natural interplay between the gill mucus metabolome and microbiome of *Chaetodon lunulatus*, as potential mechanisms involved in parasite avoidance. *C. lunulatus* is the only butterflyfish species that has never been found parasitized by gill monogeneans, despite living among parasitized butterflyfishes with similar ecology, behavior, and phylogenetic position [34]. For this reason, it provides a good model to study natural parasite evasion mechanisms.

The untargeted metabolomics study showed that parasitized and non-parasitized fish presented significantly different polar metabolome fingerprints. In a previous study, we found that butterflyfish species taxonomy was not a significant driver of metabolome variability of the polar fraction [37], suggesting other *C. lunulatus* specific traits behind these differences. The coupling of the untargeted metabolomics study with high-resolution mass spectrometry analysis allowed us to identify several metabolites, of which eight peptides (mass range between 1.2–4.5 kDa), significantly overexpressed in *C. lunulatus* gill mucus. We characterized and chemically synthesized three of these peptides (CLHbβ-1, CLHbβ-2, CLHbβ-3) found uniquely in *C. lunulatus*, which presented 100% homology to N-terminal region of β-subunit hemoglobin (Hb), suggesting that these peptides might be cleaved off from the N-terminal of Hb by specific proteases after arginine residues (i.e., trypsin-like). The other identified peptides were present in smaller quantities and could not be characterized. We also identified two additional peptides (CLHbβ-4 and CLHbβ-5) present in both parasitized and non-parasitized fish, probably resulting from the cleavage of the same protein (Hb) after leucine residues by other types of proteases (i.e., carboxypeptidase-like). Several studies have previously reported fish AMPs cleaved from functional proteins such as histones (e.g., parasin-I and hipposin) [44,45], L-amino acid oxidases [46], ribosomal proteins [47], and Hb [27]. Hb is an iron-containing oxygen-transport metalloprotein with a quaternary structure composed of two α-globin and two β-globin chains [48]. Several Hb-derived peptides with antimicrobial and antiparasitic activities have been isolated from different fish species (channel catfish *Ictalurus punctatus*, Japanese eel *Anguilla japonica,* and skipjack tuna *Katsuwonus pelamis*) and tissues (gill epithelium, liver) [27,49,50]. Interestingly, CLHbβ-1 presented some homology to Hbβ-P3, a β-chain hemoglobin peptide isolated from gill epithelium of channel catfish resulting from the same cleavage of the protein, which displays antibacterial activity [28].

Host commensal microbiota influences host physiology, playing an active role in regulating host immune homeostasis, which is crucial to avoid the proliferation of pathogens [51,52,53]. In a previous study, we characterized the core microbiota of three highly parasitized butterflyfishes and the non-parasitized *C. lunulatus* [38]. Here, we found that some bacteria typically associated with low-oxygen habitats (Fusobacteriaceae, Clostridiales), were significantly more abundant in *C. lunulatus* compared to other *Chaetodon* species. Increased expression of Hb under low-oxygen levels has been observed in organisms such as fish, plants, or humans [54,55,56]. These results suggest that *C. lunulatus* gill mucus might have lower-oxygen contents and possibly higher levels of hemoglobin than in other butterflyfish species, but this is a non-trivial analysis that will have to be developed in further studies.

Associated bacteria can also play major roles in pathogen control through the production of bioactive metabolites [12,15,57]. We have explored the correlations between metabolites and OTUs to investigate whether some bacteria are specifically related to some metabolites in *C. lunulatus*. We found that all peptides were highly correlated to at least one OTU. CLHbβ-1, CLHbβ-2, and CLHbβ-3 were highly correlated to a *Fusobacteriaceae* strain. Fusobacteria are frequently found inhabiting mucosal tissues and are known to produce a short-chain fatty acid, butyrate, which is the end-product of fermentation of carbohydrates, including those found in mucins [58]. Studies show that butyrate provides benefits to mammal hosts, enhancing mucus production and acting as anti-carcinogen and anti-inflammatory [59,60], but its effects in fish are yet unknown. If Fusobacteria enhances mucus production in fish, this could increase gill mucus thickness, increasing oxygen diffusion distance, and eventually promoting higher levels of hypoxia as previously described in clownfish [61]. Lower-oxygen levels could then promote higher hemoglobin levels, which could be cleaved by Fusobacteria or other microbial proteases (maybe for nutrition purposes), leading to the VIP peptides. Several studies have shown that extracellular microbe proteases can specifically cleave Hb and produce Hb-derived peptides with antimicrobial activities [62,63,64]. Furthermore, some studies on *Fusobacterium nucleatum*, a human pathogenic Fusobacteria, suggest peptides and amino acids are the main energy source of Fusobacteria [65,66] and several extracellular serine proteases have been isolated from *F. nucleatum* [67,68]. As CLHbβ-1, CLHbβ-2 and CLHbβ-3 are cleaved after an arginine residue, these peptides are probably produced by a trypsin-like protease, possibly from bacterial origin.

Proteases in skin mucus are involved in the natural resistance of fish to an infection either by acting directly on pathogens or by modifying the mucus consistency to allow pathogen removal [19,69]. Proteases can also activate and enhance the production of other innate immune components present in fish mucus [69]. We can suggest two hypotheses by which *C. lunulatus* specific Fusobacteriaceae could participate in monogenean control by contributing to the production of bioactive Hb-derived peptides or producing proteases acting directly on the parasite. However, findings discussed here come from different animal models and bacterial strains, and therefore more research is required to elucidate the role of Fusobacteria in *C. lunulatus* gill mucus and its putative involvement in the production of hemoglobin peptides. Equally, more studies are required to elucidate the origin (bacterial or fish) of the proteases cleaving Hb, as well as determine the putative antiparasitic activity of CLHbβ-1, CLHbβ-2, and CLHbβ-3. Since the Fusobacteriaceae OTU in *C. lunulatus* gills has no close cultivated counterpart, future metagenomic analysis of the fish mucus, and multi-metagenome assembly (e.g., [70]) could help to access the peptidases associated this OTU and test these hypotheses.

In summary, we identified and characterized three novel hemoglobin-derived peptides (CLHbβ-1, CLHbβ-2, and CLHbβ-3) exclusively found in *C. lunulatus* gill mucus using a metabolomics-microbial metabarcoding approach. We also identified specific bacterial families and OTUs typical from low-oxygen habitats in *C. lunulatus* gill mucus and found a Fusobacteriaceae strain exclusively present in *C. lunulatus* and also highly correlated to the three identified peptides. We discuss here the possible involvement of these peptides and Fusobacteriaceae to the absence of monogenean parasites in this species. Further research is in progress in order to evaluate in vitro peptide activities against butterflyfish monogeneans at different stages of their life cycle and to characterize the expression of trypsin-like proteases in the skin mucus of different fish species of this genus. In order to further study our hypothesis, the maturation process required to generate the Hb-derived peptides needs to be elucidated.

## 4. Materials and Methods 

### 4.1. Chemicals

MilliQ water (Merck Millipore, Billerica, MA, USA) was used; LC-MS-grade formic acid (FA), trifluoroacetic acid (TFA) and acetonitrile (MeCN) were from Carlo-Erba Reagents (Val de Reuil, France). All Fmoc-protected amino acids, DIC (*N,N*-diisopropylcarbodiimide), Oxyma (Ethyl cyanoglyoxylate-2-oxime), Wang resin preloaded with arginine (loading 0.29 mmol/g) or leucine (loading 0.33 mmol/g) were purchased from Iris Biotech (Marktredwitz, Germany). DCM (dichloromethane), DMF (*N,N*-dimethylformamide), cHex (Cyclohexane), TFA, TIS (Triisopropylsilane), FA, MeCN and piperidine for peptide synthesis were obtained from Sigma–Aldrich (Saint Louis, MI, USA), TIPS (triisopropyl silane, Sigma–Aldrich).

### 4.2. Animal Ethics

Animal handling and sampling protocols were pre-approved by animal experimentation experts from our institute, following the European Union directive 2010/63UE.

### 4.3. Fish Mucus Sampling

Fish mucus samples used for this study had been previously obtained, processed, and analyzed in previous works [37,38]. Briefly, the fish were spear-fished in Moorea (French Polynesia), and the mucus was collected from scrapping the gill filaments (Supplementary material S1a).

### 4.4. Study of the Gill Mucus Metabolome

#### 4.4.1. Untargeted Metabolomics

The metabolome dataset used for this study was previously obtained and described in Reverter and collaborators [37]. Briefly, the metabolome of eight butterflyfish species, *Chaetodon auriga*, *C. lunulatus*, *C. lunula*, *C. ornatissimus*, *C. quadrimaculatus*, *C. reticulatus*, *C. vagabundus,* and *C. ulietensis* was explored using a non-targeted LC-MS metabolomics pipeline. Freeze-dried mucus was extracted using a two-step biphasic extraction, which yielded polar and apolar extracts, respectively. Both extracts were analyzed using an LC-ESI-MS system, and the metabolomic data was processed using the R package XCMS, providing a matrix containing the retention time, *m*/*z* values, and integrated peak areas of the identified features (Supplementary material S1b). 

#### 4.4.2. Optimized Peptide Extraction and Peptides Purification

An acidic extraction was performed following the method described in Vidal-Dupiol et al. [71] to obtain a peptide-enriched extract. Briefly, 10 mg of freeze-dried mucus were suspended in 1 mL of 2 M glacial acetic acid. The suspension was maintained in a 4 °C water bath during sonication (Vibra- cellTM 75185, 50% power, three pulses of 30 s) and centrifuged at 10,000× *g* for 20 min at 4 °C, to remove the cellular debris. The obtained supernatant was immediately collected and prepurified by solid-phase extraction using a Strata C18 cartridge (500 mg/6 mL; Phenomenex, CA, USA) washed using acidified water (0.05% TFA), and three successive elutions were performed with 10%, 60%, and 80% acetonitrile (MeCN) in acidified water. The fractions obtained were freeze-dried and reconstituted with 1 mL of H_2_O/MeCN (1:1 *v*/*v*). *C. lunulatus* and *C. ornatissimus* mucus samples were extracted in triplicates.

#### 4.4.3. Peptide Sequence Analysis

MALDI-MS analysis of the SPE-prepurified peptides was done on a Bruker AutoFlex™ III (Bruker Daltonics, Germany). Samples were diluted 10-fold in acidified water (0.1% trifluoroacetic acid), and 0.5 μL of a given sample was mixed with 0.5 μL of 4-HCCA (Sigma–Aldrich, France) on a MALDI MTP 384 polished ground steel plate (Bruker Daltonics). Following co-crystallization, MALDI MS spectra were recorded in positive linear mode using FlexControl 4.0 software (Bruker Daltonics). The following instrument settings were used: 1.5 kV of electric potential difference, dynamic range of detection of 600 to 18,000 Da, global attenuator offset of 46% with 200 Hz laser frequency, and 2000 accumulated laser shots per sample. A suppression mass gate up to *m*/*z* 600 to prevent detector saturation by clusters of the 4-HCCA matrix. An external calibration of the mass spectrometer was performed using a standard mixture of peptides and proteins (Peptide Standard Calibration II and Protein Standard Calibration I, Bruker Daltonics) covering the dynamic range of analysis.

NanoLC-MS/MS analyses were carried out using an Ultimate 3000 nano-HPLC coupled with a Q-Exactive Orbitrap mass spectrometer (Thermo Fisher Scientific, MA, USA). The peptides were loaded onto a C18 PepMap100 precolumn (5 μm, 300 μm × 5 mm) at 10 μL·min^−1^, and separated onto an Acclaim C18 PepMap100 column (75 μm × 250 mm, 3 μm 100 A) at 300 nL min^−1^, both maintained at 35 °C (both columns from Thermo Fisher Scientific). Peptide samples were eluted by a gradient of H_2_O/MeCN (A/B), both containing 0.1% formic acid, from 2% to 50% B in 31 min. For MS analysis, the Q-Exactive mass spectrometer was used in positive mode and data-dependent acquisition. The voltage applied to the nano-tips was adjusted to produce 0.3 μA and the entrance capillary was maintained at 320 °C. The Q-Exactive Orbitrap acquired a full-range scan from 380 to 2000 *m*/*z* at 70,000 resolution, automatic gain control (AGC) target 3·10^6^, maximum ion trap time (IT) 200 ms, and then fragmented the top ten-peptide ions in each cycle (range 200–2000 *m*/*z*, 17,500 resolution, AGC target 2·10^5^, maximum IT 100 ms, intensity threshold 4·10^4^, excluding charge-unassigned ions, normalised collision energy at 27). Parent ions were then excluded from MS/MS for the next 10 s. The software Chromeleon Xpress and Xcalibur 2.2 (Thermo Fisher Scientific) were used to control the HPLC and the mass spectrometer, respectively. Exact masses were calculated with the Xtract algorithm from Xcalibur software.

Characterization of peptide sequences was performed by Proteome Discoverer 1.4 (Thermo Fisher Scientific). Homology searches of the amino acid sequence were performed on the Swissprot-UniprotKB database (November 2016, https://www.uniprot.org/) using Mascot and Sequest.

#### 4.4.4. Solid-Phase Synthesis and Peptide Analysis

Peptides were synthesized by solid-phase peptide synthesis (SPPS) using Fmoc/O-tbutyl chemistry on an automated microwave peptide synthesizer (CEM liberty one, Orsay, France). SPPS was performed at a 0.10 mmol scale with DIC/Oxyma as a coupling reagent and 20% piperidine in DMF for Fmoc deprotection. The elongation was carried out automatically using a 5-fold excess of protected amino acids and coupling reagent. The mixture was irradiated in a microwave cavity at 70 °C for 20 min. Fmoc deprotection was achieved using 20% piperidine in DMF (7 mL for 30 s at 33 °C and 7 mL for 3 min at 70 °C). After completion of the synthesis, the peptidyl-resin was washed with 2 × 10 mL of DCM. Cleavage of the peptide and removal of the protecting groups were performed by treating the peptidyl-resin with 10 mL of TFA/DCM/H_2_O/TIPS 4.7:4.7:4:2 (*v*/*v*) for 60 min. After the resins’ filtration, the filtrate was concentrated and co-evaporated with cyclohexane. The peptide was precipitated with diethyl ether and recovered by centrifugation. Following purification, peptide purity was established by HPLC-ELSD analysis, and identity was confirmed by comparison of either retention times and spectral data of natural peptides using UHPLC-HRMS/MS. These analyses were performed on a UHPLC system (Vanquish, Thermo Scientific) interfaced with an Orbitrap mass spectrometer (Q-Exactive Plus Hybrid, Thermo Scientific) using a Luna Omega Polar C18 column (2.1 mm × 100 mm, 1.6 µm particle size, 100 A porosity, Phenomenex). The elution rate was set to 0.3 mL·min^−1^ at a constant temperature of 30 °C. The elution was performed using a gradient of H_2_O/MeCN (A/B), both containing 0.1% formic acid, from 2% to 50% B in 11 min. Mass spectrometer parameters were set as follows: spray voltage 3500 V, capillary temperature 320 °C, sheath gas N_2_ pressure 35 (arbitrary unit), auxiliary gas pressure 10 (arbitrary unit). Mass spectrometric data were collected in continuum full-range scan mode from *m*/*z* 300–1000 in positive mode with a resolution of 70,000. 

**CLHbβ-1:** LLIVYPWTQR Crude peptide (152 mg, 100% yield) was purified by semi-preparative HPLC using a linear gradient from 10% to 100% B in A over 50 min with a flow rate of 5 mL/min to yield CLHbβ-1 (55 mg, 42% yield, > 98% purity). ESI-HRMS: Calculated for C_63_H_97_N_15_O_14_: [M + H]^+^ *m*/*z* 1288.74177; [M + 2H]^2+^ *m*/*z* 644.87479; [M + 3H]^3+^ *m*/*z* 430.1914; found *m*/*z*: 644.8743.

**CLHbβ-2:** VKWTDAERAAITSLWGKIDVGEIGPQALTR Crude peptide (268 mg, 71% yield, > 90% purity). ESI-HRMS: Calculated for C_147_H_237_N_41_O_44_: [M+H]^+^ *m*/*z* 3281.76464; [M + 2H]^2+^ *m*/*z* 1641.38623; [M + 3H]^3+^ *m*/*z* 1094.5934; [M + 4H]^4+^ *m*/*z* 821.19703; [M + 5H]^5+^ *m*/*z* 657.1592 found *m*/*z*: 657.1589.

**CLHbβ-3:** AAITSLWGKIDVGEIGPQALTR Crude peptide (210 mg, 83% yield, > 90% purity). ESI-HRMS: Calculated for C_103_H_170_N_28_O_31_: [M + H]^+^ *m*/*z* 2296.26651; [M + 2H]^2+^ *m*/*z* 1148.6372; [M + 3H]^3+^ *m*/*z* 766.0941; found *m*/*z*: 766.0927.

**CLHbβ-4:** VKWTDAERAAITSLWGKIDVGEIGPQALTRL Crude peptide (220 mg, 65% yield, > 90% purity). ESI-HRMS: Calculated for C_153_H_248_N_42_O_45_: [M + H]^+^ *m*/*z* 3394.84871; [M + 2H]^2+^ *m*/*z* 1697.92827; [M + 3H]^3+^ *m*/*z* 1132.28757; [M + 4H]^4+^ *m*/*z* 849.46802; [M + 5H]^5+^ *m*/*z* 679.77600 found *m*/*z*: 679.7773.

**CLHbβ-5:** VKWTDAERAAITSLWGKIDVGEIGPQALTRLL Crude peptide (257 mg, 83% yield, > 90% purity). ESI-HRMS: Calculated for C_159_H_259_N_43_O_46_: [M + H]^+^ *m*/*z* 3507.93277; [M + 2H]^2+^ *m*/*z* 1754.47029; [M + 3H]^3+^ *m*/*z* 1169.98281; [M + 4H]^4+^ *m*/*z* 877.73906; [M + 5H]^5+^ *m*/*z* 702.3928 found *m*/*z*: 702.3931.

### 4.5. Study of the Gill Mucus Associated Bacterial Communities

The bacterial community dataset was previously acquired and described in Reverter and collaborators [38]. Briefly, the bacterial communities of gill mucus of four butterflyfish species (*C. lunulatus*, *C. ornatissimus*, *C. reticulatus*, and *C. vagabundus*) were studied. Gill mucus DNA was extracted, and DNA samples were sent for 454 pyrosequencing. Multiplex raw SFF (standard flowgram format) files were analyzed using a hybrid analysis pipeline using Usearch5 and Qiime1, and non-chimeric sequences were unweighted and grouped into operational taxonomic units (OTUs) with a cut-off of 98% (Supplementary material S1c); this provided a rarefied table containing the relative abundance (%) of 1041 OTUs from 14 butterflyfish gill mucus samples. 

### 4.6. Data analysis

Multidimensional matrices obtained from the metabolome and microbiome data were analyzed equally. Principal coordinate analysis (PCoA, library ape for R) was used to visualize the differences between the parasitized and non-parasitized fish metabolome and microbiome. Analysis of similarities (ANOSIM, 10,000 permutations, library vegan for R) was used to detect significant differences between the PCoA groups. Partial least squares discriminant analysis (PLS-DA, library mixOmics for R) was used to select the metabolites and OTUs driving the differences between the two fish groups. PLS-DA model accuracy was calculated using two-fold cross-validation (2CV, function MVA.cmv from package RVAideMemoire for R) and the resulting number of misclassifications (NMC) was compared to its permutated null distribution (999 permutations) to test for model significance (*p*-value < 0.05, function MVA.test from package RVAideMemoire for R). Variable importance in projection (VIP) was obtained (package RVAiedeMemoire for R), and features and OTUs with VIP scores higher than 1 were retained. Kruskal–Wallis test was used to test for a significant difference in the intensity of metabolites and relative abundance of OTUs between parasitized and non-parasitized fish. In order to explore the correlation between VIP metabolites and OTUs, a correlation network was constructed using the software Cytoscape v3.4.0 [72]. Spearman correlations between the VIP metabolite and OTUs were computed, and lines were drawn between pairs of metabolites (circular nodes) and OTUs (diamond nodes) when their correlation was higher than 0.5.

## Figures and Tables

**Table 1:** Sequences and mass spectrometric data for peptides CLHbβ-1 to 5.

Name	Sequence	Calculated MW (Monoisotopic Mass)	*m*/*z* LC-MS (Monoisotopic Mass)				*m*/*z* MALDI-MS (Average Value)	
			Charge State	Calculated *m*/*z* LC-MS	Observed for Natural Peptides	Observed for Synthetic Peptides	Theoretical MH+	Observed MH+
CLHbβ-2	LLIVYPWTQR	1287.7339	2	644.8748	644.8738	644.8743	1289.562	1289.682
CLHbβ-3	AAITSLWGKIDVGEIGPQALTR	2295.2586	3	766.0940	766.0947	766.0927	2297.659	2296.555
CLHbβ-1	VKWTDAERAAITSLWGKIDVGEIGPQALTR	3280.7567	5	657.1592	657.1599	657.1589	3283.754	3283.980
CLHbβ-4	VKWTDAERAAITSLWGKIDVGEIGPQALTRL	3393.8408	5	679.7760	679.7758	679.7773	3396.914	3396.935
CLHbβ-5	VKWTDAERAAITSLWGKIDVGEIGPQALTRLL	3506.9248	5	702.3928	702.3929	702.3931	3510.073	3510.192

## Supplementary Materials

The following are available online at https://www.mdpi.com/2218-1989/10/6/227/s1, Figure S1: PCoA analysis of gill mucus metabolome (apolar fraction) of parasitized (other, orange triangles) and nonparasitized (*C. lunulatus*, CL, blue circles) butterflyfish. ANOSIM *p*-value > 0.05, Figure S2: LC-HRMS ESI+ analysis of the synthetic peptide CLHbβ-1 and the acidic enriched peptidic fraction of *C. lunulatus* mucus, Figure S3: LC-HRMS ESI+ analysis of the synthetic peptide CLHbβ-2 and the acidic enriched peptidic fraction of *C. lunulatus* mucus, Figure S4: LC-HRMS ESI+ analysis of the synthetic peptide CLHbβ-3 and the acidic enriched peptidic fraction of *C. lunulatus* mucus in ion extraction mode, Figure S5: LC-HRMS ESI+ analysis of the synthetic peptide CLHbβ-4 and the acidic enriched peptidic fraction of *C. lunulatus* mucus, Figure S6: LC-HRMS ESI+ analysis of the synthetic peptide CLHbβ-5 and the acidic enriched peptidic fraction of *C. lunulatus* mucus, Figure S7: MALDI-MS spectrum of the peptides extracted from *C. lunulatus* gill mucus, Figure S8: MS-MS spectrum of the peptides CLHbβ #1 to 5, Table S1: Acid-extract-nanoLC-MS-MS protein identification report. Supplementary material S1: Details on the material and method section related to previously published work.

## References

[1] Hudson P.J., Dobson A.P., Lafferty K.D. (2006). Is a healthy ecosystem one that is rich in parasites?. Trends Ecol. Evol..

[2] Fox N.J., Marion G., Davidson R., White P.C.L., Hutchings M. (2015). Climate-driven tipping-points could lead to sudden, high-intensity parasite outbreaks. R. Soc. Open Sci..

[3] Cable J., Barber I., Boag B., Ellison A.R., Morgan E.R., Murray K.A., Pascoe E.L., Sait S.M., Wilson A., Booth M. (2017). Global change, parasite transmission and disease control: Lessons from ecology. Philos. Trans. R. Soc. B.

[4] Perry B.D., Grace D., Sones K. (2011). Current drivers and future directions of global livestock disease dynamics. Proc. Natl. Acad. Sci. USA.

[5] Sana S., A Hardouin E., E Gozlan R., Ercan D., Tarkan A.S., Zhang T., Andreou D. (2017). Origin and invasion of the emerging infectious pathogen *Sphaerothecum destruens*. Emerg. Microbes Infect..

[6] Cunningham A.A., Dobson A.P., Hudson P.J. (2012). Disease invasion: Impacts on biodiversity and human health. Philos. Trans. R. Soc. B.

[7] Rakers S., Niklasson L., Steinhagen D., Kruse C., Schauber J., Sundell K., Paus R. (2013). Antimicrobial Peptides (AMPs) from Fish Epidermis: Perspectives for Investigative Dermatology. J. Investig. Dermatol..

[8] Hahn M.A., Dheilly N.M. (2016). Experimental Models to Study the Role of Microbes in Host-Parasite Interactions. Front. Microbiol..

[9] Quigley B.J.Z., López D.G., Buckling A., McKane A.J., Brown S. (2012). The mode of host–parasite interaction shapes coevolutionary dynamics and the fate of host cooperation. Proc. R. Soc. B.

[10] Dheilly N.M., Poulin R., Thomas F. (2015). Biological warfare: Microorganisms as drivers of host–parasite interactions. Infect. Genet. Evol..

[11] Cirimotich C., Dong Y., Clayton A.M., Sandiford S.L., Souza-Neto J.A., Mulenga M., Dimopoulos G. (2011). Natural Microbe-Mediated Refractoriness to *Plasmodium* Infection in *Anopheles Gambiae*. Science.

[12] Lowrey L., Woodhams D.C., Tacchi L., Salinas I. (2015). Topographical Mapping of the Rainbow Trout (*Oncorhynchus mykiss*) Microbiome Reveals a Diverse Bacterial Community with Antifungal Properties in the Skin. Appl. Environ. Microbiol..

[13] Stevens J., Jackson R., Olson J. (2016). Bacteria associated with lionfish (*Pterois volitans*/miles complex) exhibit antibacterial activity against known fish pathogens. Mar. Ecol. Prog. Ser..

[14] Gómez G.D., Balcázar J.L. (2008). A review on the interactions between gut microbiota and innate immunity of fish: Table 1. FEMS Immunol. Med. Microbiol..

[15] Sepahi A., Cordero H., Goldfine H., Esteban M. (2016). Ángeles; Salinas, I. Symbiont-derived sphingolipids modulate mucosal homeostasis and B cells in teleost fish. Sci. Rep..

[16] Reverter M., Tapissier-Bontemps N., Lecchini D., Banaigs B., Sasal P. (2018). Biological and Ecological Roles of External Fish Mucus: A Review. Fishes.

[17] Hansson G.C. (2011). Role of mucus layers in gut infection and inflammation. Curr. Opin. Microbiol..

[18] Gómez D., Sunyer J.O., Salinas I. (2013). The mucosal immune system of fish: The evolution of tolerating commensals while fighting pathogens. Fish Shellfish Immunol..

[19] Kallert D.M., Bauer W., Haas W., El-Matbouli M. (2011). No shot in the dark: Myxozoans chemically detect fresh fish. Int. J. Parasitol..

[20] Brooker A.J., Shinn A.P., Souissi S., Bron J.E. (2013). Role of kairomones in host location of the pennellid copepod parasite, *Lernaeocera branchialis* (L. 1767). Parasitology.

[21] Ohashi H., Umeda N., Hirazawa N., Ozaki Y., Miura C., Miura T. (2007). Purification and identification of a glycoprotein that induces the attachment of oncomiracidia of *Neobenedenia girellae* (Monogenea, Capsalidae). Int. J. Parasitol..

[22] Igarashi K., Matsunaga R., Hirakawa S., Hosoya S., Suetake H., Kikuchi K., Suzuki Y., Nakamura O., Miyadai T., Tasumi S. (2017). Mucosal IgM Antibody with d-Mannose Affinity in Fugu *Takifugu rubripes* Is Utilized by a Monogenean Parasite *Heterobothrium okamotoi* for Host Recognition. J. Immunol..

[23] Hellio C., Pons A.M., Beaupoil C., Bourgougnon N., Le Gal Y. (2002). Antibacterial, antifungal and cytotoxic activities of extracts from fish epidermis and epidermal mucus. Int. J. Antimicrob. Agents.

[24] Wang H., Tang W., Zhang R., Ding S. (2019). Analysis of enzyme activity, antibacterial activity, antiparasitic activity and physico-chemical stability of skin mucus derived from *Amphiprion clarkii*. Fish Shellfish Immunol..

[25] Masso-Silva J.A., Diamond G. (2014). Antimicrobial Peptides from Fish. Pharmaceuticals.

[26] Colorni A., Ullal A., Heinisch G., Noga E.J. (2008). Activity of the antimicrobial polypeptide piscidin 2 against fish ectoparasites. J. Fish Dis..

[27] Ullal A., Litaker R.W., Noga E.J. (2008). Antimicrobial peptides derived from hemoglobin are expressed in epithelium of channel catfish (*Ictalurus punctatus*, Rafinesque). Dev. Comp. Immunol..

[28] Ullal A.J., Noga E.J. (2010). Antiparasitic activity of the antimicrobial peptide HbbetaP-1, a member of the beta-haemoglobin peptide family. J. Fish Dis..

[29] Plaisance L., Bouamer S., Morand S. (2004). Description and redescription of *Haliotrema* species (Monogenoidea: Poloyonchoinea: Dactylogyridae) parasitizing butterfly fishes (Teleostei: Chaetodontidae) in the Indo-West Pacific Ocean. Parasitol. Res..

[30] Kearn G. (1994). Evolutionary expansion of the Monogenea. Int. J. Parasitol..

[31] Frantz A., Perga M.-E., Guillard J. (2018). Parasitic versus nutritional regulation of natural fish populations. Ecol. Evol..

[32] Dash P., Kar B., Mishra A., Sahoo P.K. (2014). Effect of *Dactylogyrus catlaius* (Jain 1961) infection in *Labeo rohita* (Hamilton 1822): Innate immune responses and expression profile of some immune-related genes. Indian J. Exp. Biol..

[33] Li J.-P., Fu Y.-W., Zhang Q.-Z., Xu D.-H., Lin Y.-M., Zhou S.-Y., Lin D.-J. (2018). Grass carp which survive *Dactylogyrus ctenopharyngodonid* infection also gain partial immunity against *Ichthyophthirius multifili*. Dis. Aquat. Org..

[34] Reverter M., Cutmore S.C., Bray R., Cribb T.H., Sasal P. (2016). Gill monogenean communities (Platyhelminthes, Monogenea, Dactylogyridae) of butterflyfishes from tropical Indo-West Pacific Islands. Parasitology.

[35] Pratchett M.S. (2005). Dietary overlap among coral-feeding butterflyfishes (Chaetodontidae) at Lizard Island, northern Great Barrier Reef. Mar. Biol..

[36] Bellwood D.R., Klanten S., Cowman P.F., Pratchett M.S., Konow N., Van Herwerden L. (2010). Evolutionary history of the butterflyfishes (f: Chaetodontidae) and the rise of coral feeding fishes. J. Evol. Biol..

[37] Reverter M., Sasal P., Banaigs B., Lecchini D., Lecellier G., Tapissier-Bontemps N. (2017). Fish mucus metabolome reveals fish life-history traits. Coral Reefs.

[38] Reverter M., Sasal P., Tapissier-Bontemps N., Lecchini D., Suzuki M. (2017). Characterisation of the gill mucosal bacterial communities of four butterflyfish species: A reservoir of bacterial diversity in coral reef ecosystems. FEMS Microbiol. Ecol..

[39] Dibattista J.D., Wang X., Saenz-Agudelo P., Piatek M.J., Aranda M., Berumen M.L. (2016). Draft genome of an iconic Red Sea reef fish, the blacktail butterflyfish (*Chaetodon austriacus*): Current status and its characteristics. Mol. Ecol. Resour..

[40] Shinn A., Pratoomyot J., Bron J., Paladini G., Brooker E., Brooker A. Economic Impacts of Aquatic Parasites on Global Finfish Production. https://www.aquaculturealliance.org/advocate/economic-impacts-of-aquatic-parasites-on-global-finfish-production/.

[41] Picón-Camacho S.M., Marcos-Lopez M., Bron J.E., Shinn A. (2011). An assessment of the use of drug and non-drug interventions in the treatment of *Ichthyophthirius multifiliis* Fouquet, 1876, a protozoan parasite of freshwater fish. Parasitology.

[42] Forwood J.M., Harris J., DeVeney M.R. (2013). Efficacy of current and alternative bath treatments for *Lepidotrema bidyana* infecting silver perch, *Bidyanus bidyanus*. Aquaculture.

[43] Reverter M., Sarter S., Caruso D., Avarre J.-C., Combe M., Pepey E., Pouyaud L., Vega-Heredía S., De Verdal H., Gozlan R.E. (2020). Aquaculture at the crossroads of global warming and antimicrobial resistance. Nat. Commun..

[44] Birkemo G.A., Lüders T., Andersen Ø., Nissen-Meyer J. (2003). Hipposin, a histone-derived antimicrobial peptide in Atlantic halibut (*Hippoglossus hippoglossus* L.). Biochim. Biophys. Acta (BBA) - Bioenerg..

[45] Park I.Y., Park C.B., Kim M.S., Kim S.C. (1998). Parasin I, an antimicrobial peptide derived from histone H2A in the catfish, *Parasilurus asotus*. FEBS Lett..

[46] Salles C.M.C., Gagliano P., Leitão S.A.T., Salles J.B., Guedes H.L.D.M., Cassano V.P.F., De Simone S.G. (2007). Identification and characterization of proteases from skin mucus of tambacu, a Neotropical hybrid fish. Fish Physiol. Biochem..

[47] Fernandes J.M., Smith V.J. (2002). A novel antimicrobial function for a ribosomal peptide from rainbow trout skin. Biochem. Biophys. Res. Commun..

[48] Anthea M. (1993). Human Biology and Health.

[49] Zhang D.L., Rui-Zhang G., Huang W.S., Xiong J. (2013). Isolation and characterization of a novel antibacterial peptide derived from hemoglobin alpha in the liver of Japanese eel, *Anguilla japonica*. Fish Shellfish Immunol..

[50] Seo J.-K., Lee M.J., Jung H.-G., Go H.-J., Kim Y.J., Park N.G. (2014). Antimicrobial function of SHβAP, a novel hemoglobin β chain-related antimicrobial peptide, isolated from the liver of skipjack tuna, *Katsuwonus pelamis*. Fish Shellfish. Immunol..

[51] Wu H.-J., Wu E. (2012). The role of gut microbiota in immune homeostasis and autoimmunity. Gut Microbes.

[52] Boutin S., Audet C., Derome N. (2013). Probiotic treatment by indigenous bacteria decreases mortality without disturbing the natural microbiota of *Salvelinus fontinalis*. Can. J. Microbiol..

[53] Boutin S., Sauvage C., Bernatchez L., Audet C., Derome N. (2014). Inter Individual Variations of the Fish Skin Microbiota: Host Genetics Basis of Mutualism?. PLoS ONE.

[54] Gaulke G.L., Dennis C.E., Wahl D.H., Suski C.D. (2013). Acclimation to a low oxygen environment alters the hematology of largemouth bass (*Micropterus salmoides*). Fish Physiol. Biochem..

[55] Heinicke K., Prommer N., Cajigal J., Viola T., Behn C., Schmidt W. (2003). Long-term exposure to intermittent hypoxia results in increased hemoglobin mass, reduced plasma volume, and elevated erythropoietin plasma levels in man. Graefe’s Arch. Clin. Exp. Ophthalmol..

[56] Hunt P.W., Klok E.J., Trevaskis B., Watts R.A., Ellis M.H., Peacock W.J., Dennis E.S. (2002). Increased level of hemoglobin 1 enhances survival of hypoxic stress and promotes early growth in *Arabidopsis thaliana*. Proc. Natl. Acad. Sci. USA.

[57] Sanchez L.M., Wong W.R., Riener R.M., Schulze C.J., Linington R.G. (2012). Examining the Fish Microbiome: Vertebrate-Derived Bacteria as an Environmental Niche for the Discovery of Unique Marine Natural Products. PLoS ONE.

[58] Bennett K.W., Eley A. (1993). *Fusobacteria*: New taxonomy and related diseases. J. Med. Microbiol..

[59] Von Engelhardt W., Bartels J., Kirschberger S., Zu Düttingdorf H.M., Busche R. (1998). Role of short-chain fatty acids in the hind gut. Veter- Q..

[60] Andoh A., Bamba T., Sasaki M. (1999). Physiological and Anti-Inflammatory Roles of Dietary Fiber and Butyrate in Intestinal Functions. J. Parenter. Enter. Nutr..

[61] Hess S., Wenger A.S., Ainsworth T.D., Rummer J.L. (2015). Exposure of clownfish larvae to suspended sediment levels found on the Great Barrier Reef: Impacts on gill structure and microbiome. Sci. Rep..

[62] Jiang N., Tan N.S., Ho B., Ding J.L. (2007). Respiratory protein–generated reactive oxygen species as an antimicrobial strategy. Nat. Immunol..

[63] Du R., Ho B., Ding J.L. (2009). Rapid reprogramming of haemoglobin structure-function exposes multiple dual-antimicrobial potencies. EMBO J..

[64] Lee S.K., Ding J.L. (2013). A Perspective on the Role of Extracellular Hemoglobin on the Innate Immune System. DNA Cell Biol..

[65] Bakken V., Högh B.T., Jensen H.B. (1989). Utilization of amino acids and peptides by Fusobacterium nucleatum. Eur. J. Oral Sci..

[66] Rogers A.H., Gully N.J., Pfennig A.L., Zilm P.S. (1992). The breakdown and utilization of peptides by strains of *Fusobacterium nucleatum*. Oral Microbiol. Immunol..

[67] Brokstad K.A., Jensen H.B. (1991). Purification and characterization of a 65-kilodalton diisopropylfluorophosphate-binding protein in the outer membrane of *Fusobacterium nucleatum* Fev1. Eur. J. Oral Sci..

[68] Doron L., Coppenhagen-Glazer S., Ibrahim Y., Eini A., Naor R., Rosen G., Bachrach G. (2014). Identification and Characterization of Fusolisin, the *Fusobacterium nucleatum* Autotransporter Serine Protease. PLoS ONE.

[69] Esteban M. (2012). Ángeles An Overview of the Immunological Defenses in Fish Skin. ISRN Immunol..

[70] Albertsen M., Hugenholtz P., Skarshewski A., Nielsen K.L., Tyson G.W., Nielsen P.H. (2013). Genome sequences of rare, uncultured bacteria obtained by differential coverage binning of multiple metagenomes. Nat. Biotechnol..

[71] Vidal-Dupiol J., Ladrière O., Destoumieux-Garzón D., Sautiere P.-E., Meistertzheim A.-L., Tambutté E., Tambutté S., Duval D., Fouré L., Adjeroud M. (2011). Innate Immune Responses of a Scleractinian Coral to Vibriosis*. J. Biol. Chem..

[72] Shannon P., Markiel A., Ozier O., Baliga N.S., Wang J.T., Ramage D., Amin N., Schwikowski B., Ideker T. (2003). Cytoscape: A Software Environment for Integrated Models of Biomolecular Interaction Networks. Genome Res..

