# Supplementary material for: Insights into the Natural Defenses of a Coral Reef Fish Against Gill Ectoparasites: Integrated Metabolome and Microbiome Approach
Source: Metabolites. 2020 May 30;10(6):227. doi: 10.3390/metabo10060227 (PMC7345202; doi:10.3390/metabo10060227)
Supplement: Supplementary file 1 [file metabolites-10-00227-s001.zip › supplementary/Supplementary Data-Reverter et al..pdf]

Supplementary data

Insights into the natural defences of a coral reef fish against gill ectoparasites: integrated metabolome and microbiome approache.

Reverter et al.

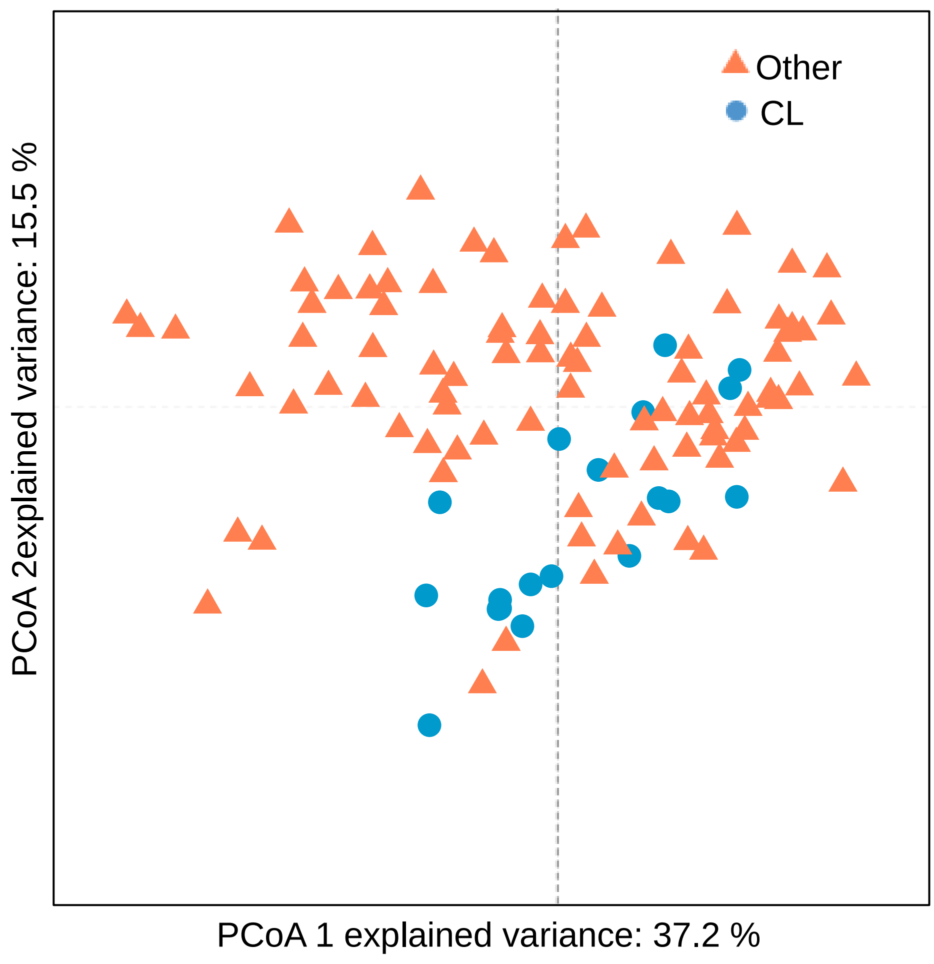

Supplementary Figure 1: PCoA analysis of gill mucus metabolome (apolar fraction) of parasitized (other, orang triangles) and non-parasitized (C. lunulatus, CL, blue circles) butterflyfish. ANOSIM P-value > 0.05.

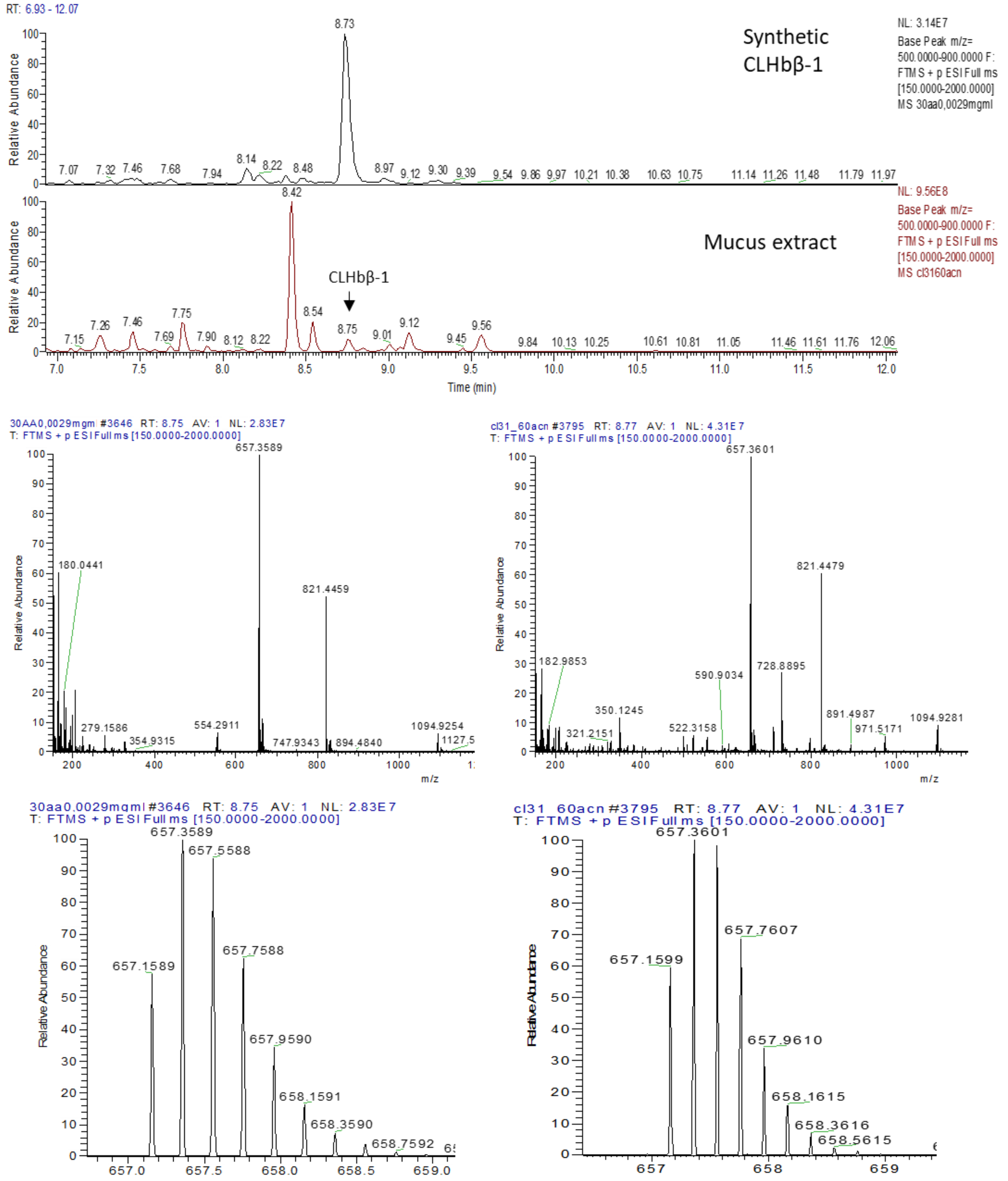

Supplementary Figure 2: LC-HRMS ESI^{+} analysis of the synthetic peptide CLHbβ-1 and the acidic enriched peptidic fraction of *Chaetodon lunulatus* mucus. Respective mass spectrum and isotopic cluster of multicharged ions of synthetic (left) and natural (right) peptides are presented below the chromatograms.

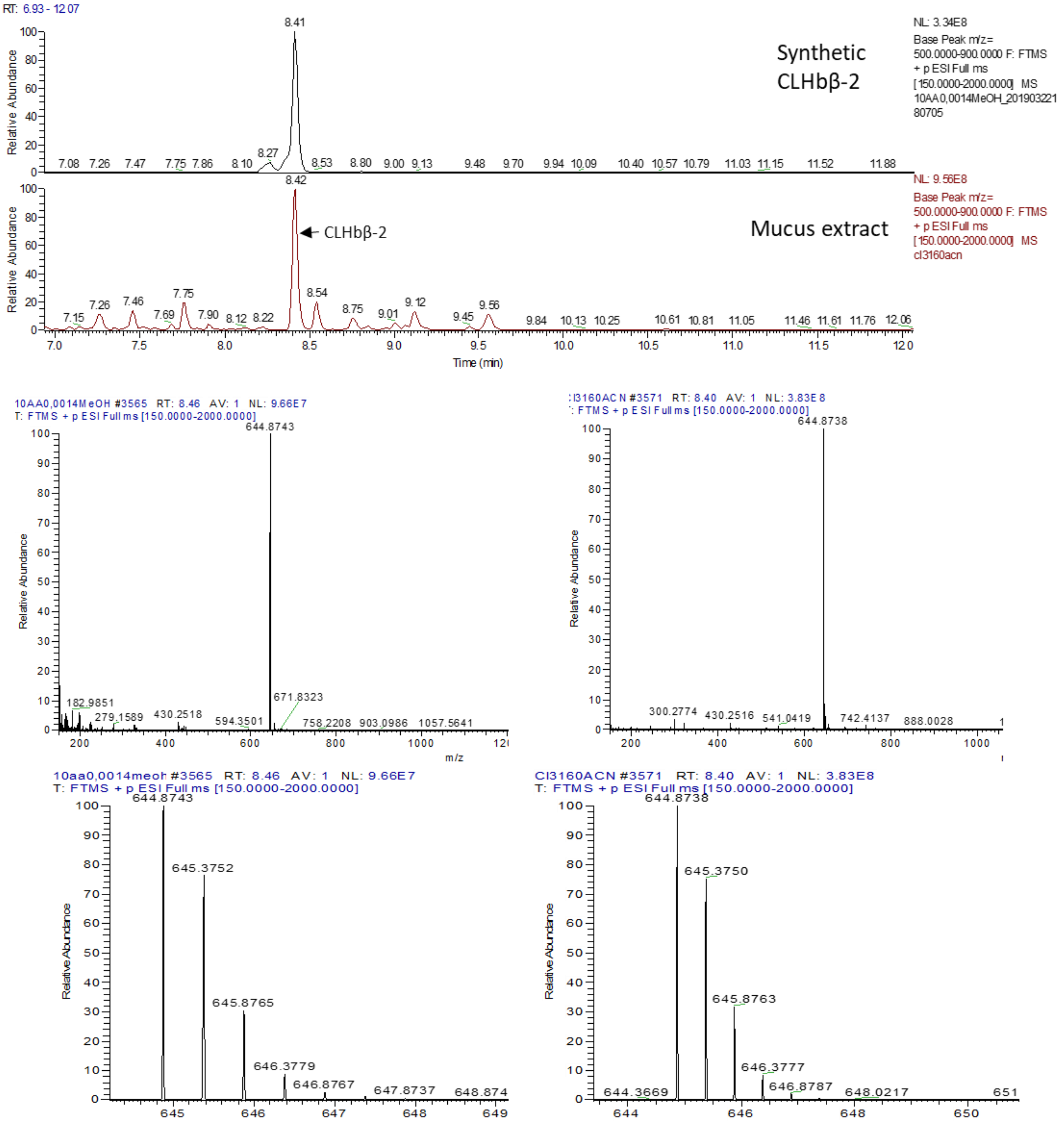

Supplementary Figure 3: LC-HRMS ESI+ analysis of the synthetic peptide CLHbβ-2 and the acidic enriched peptidic fraction of *Chaetodon. lunulatus* mucus. Respective mass spectrum and isotopic cluster of multicharged ions of synthetic (left) and natural (right) peptides are presented below the chromatograms.

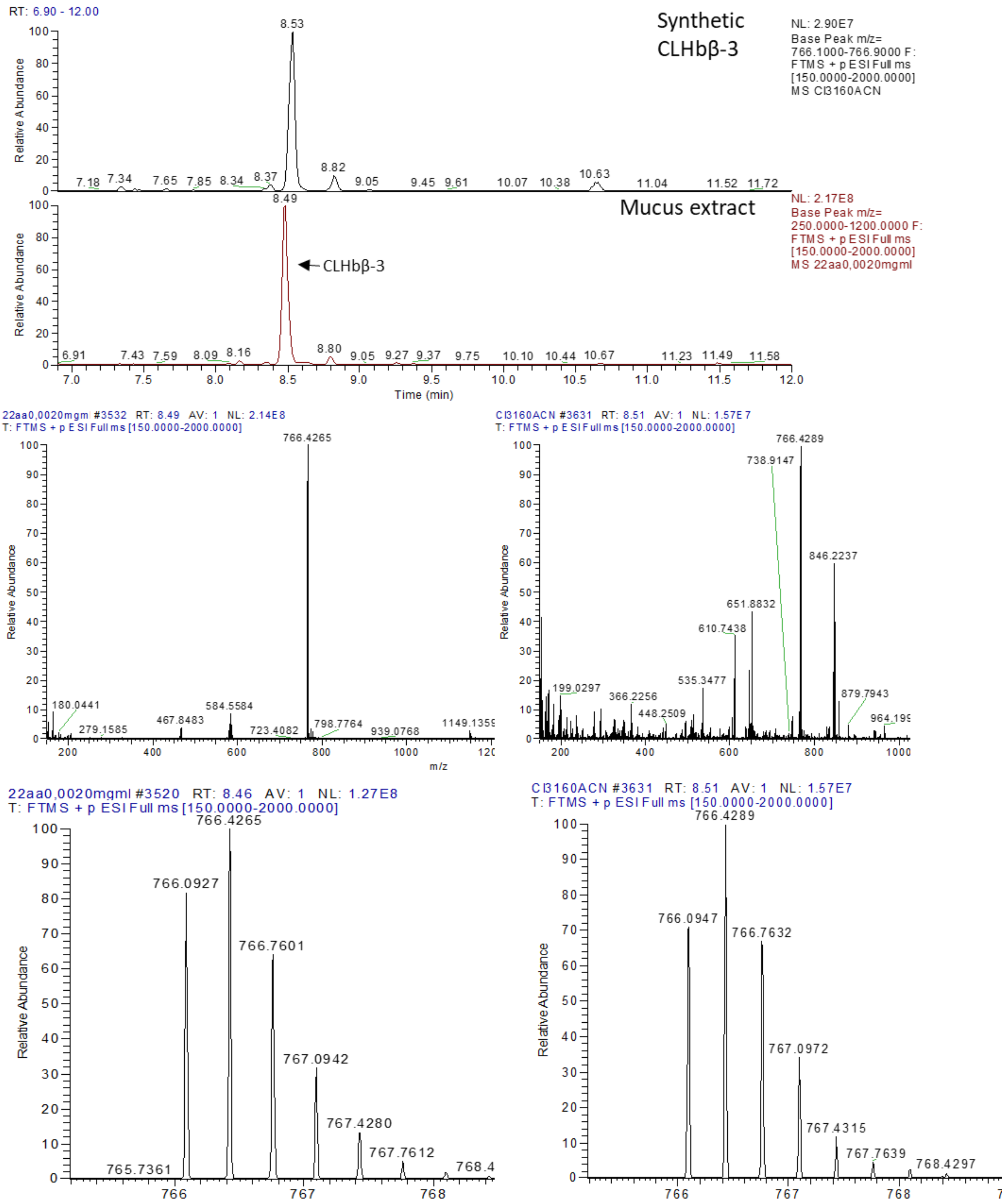

Supplementary Figure 4: LC-HRMS ESI+ analysis of the synthetic peptide CLHbβ-3 and the acidic enriched peptidic fraction of *Chaetodon lunulatus* mucus in ion extraction mode. Respective mass spectrum and isotopic cluster of multicharged ions of synthetic (left) and natural (right) peptides are presented below the chromatograms.

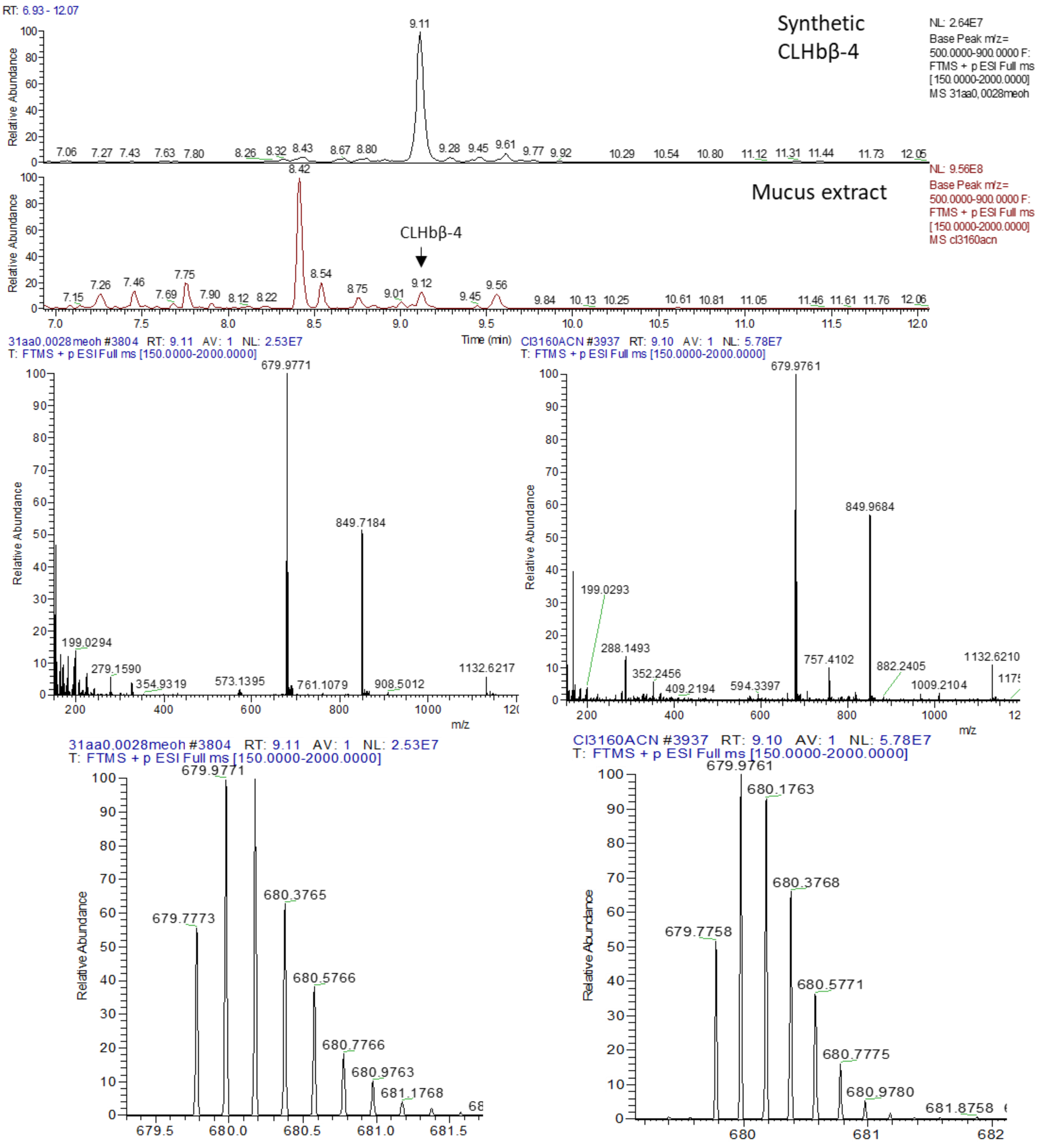

Supplementary Figure 5: LC-HRMS ESI+ analysis of the synthetic peptide CLHbβ-4 and the acidic enriched peptidic fraction of *Chaetodon lunulatus* mucus. Respective mass spectrum and isotopic cluster of multicharged ions of synthetic (left) and natural (right) peptides are presented below the chromatograms.

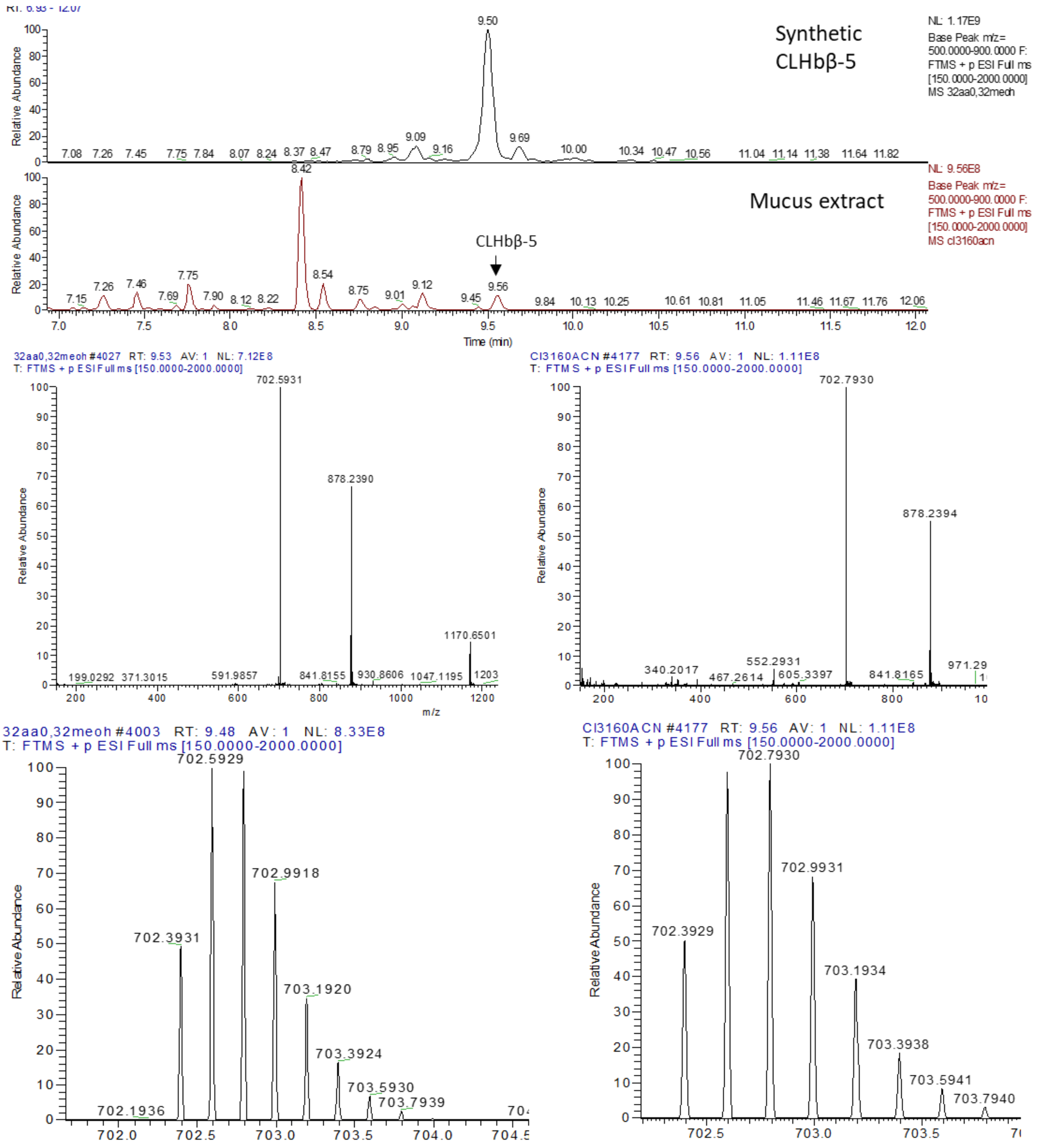

Supplementary Figure 6: LC-HRMS ESI+ analysis of the synthetic peptide CLHbβ-5 and the acidic enriched peptidic fraction of *Chaetodon lunulatus* mucus. Respective mass spectrum and isotopic cluster of multicharged ions of synthetic (left) and natural (right) peptides are presented below the chromatograms.

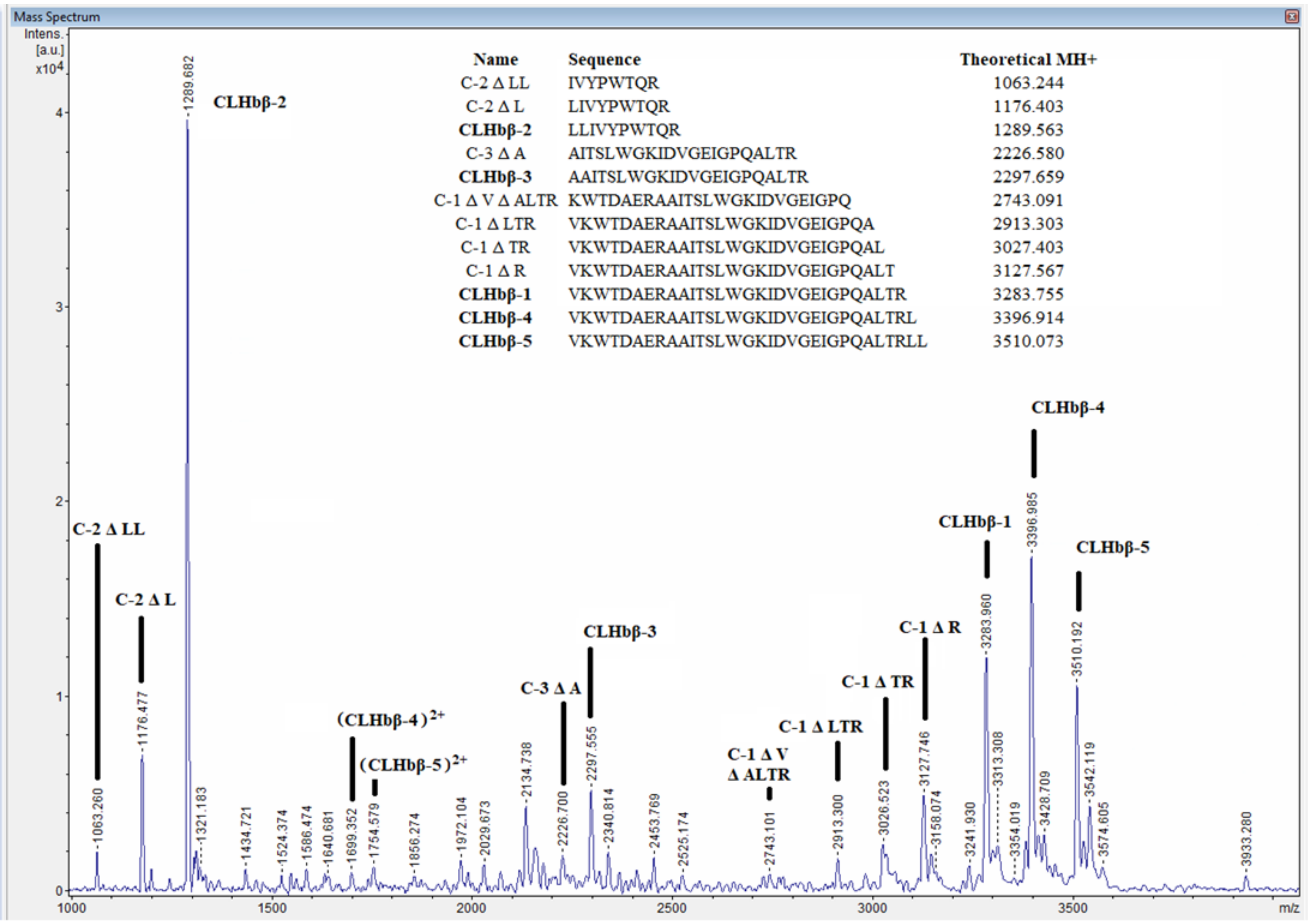

Supplementary Figure 7: MALDI-MS spectrum of the peptides extracted from *Chaetodon lunulatus* gill mucus

Peptides from the gill mucus of the butterflyfish *C. lunulatus* were acid-extracted and pre-fractionated by C_{18} solid-phase extraction (SPE). The displayed spectrum is the result of the MALDI-MS analysis in positive linear mode of the fraction eluted with 60% MeCN in 0.05% TFA water (see Material-Method for details). The table inserted in the spectrum is listing the sequences and theoretical *m/z* values (average mass) of the MH^+ ion of the five peptides which were further analysed in this study (CLHbβ #1 to 5). This MALDI-MS spectrum also reveals the presence of multiple hypothetical peptides which, according to the mass differences, derive from the degradation of the CLHbβ-1, CLHbβ-2 or CLHbβ-3 peptide (peaks labeled C-1 Δ X, C-2 Δ X, or C-3 Δ X, respectively, X being the aminoacids lost from the N-terminal or C-terminal ends). In case of CLHbβ 4 and -5, peaks corresponding to the double –charged ions (2+) are also present.

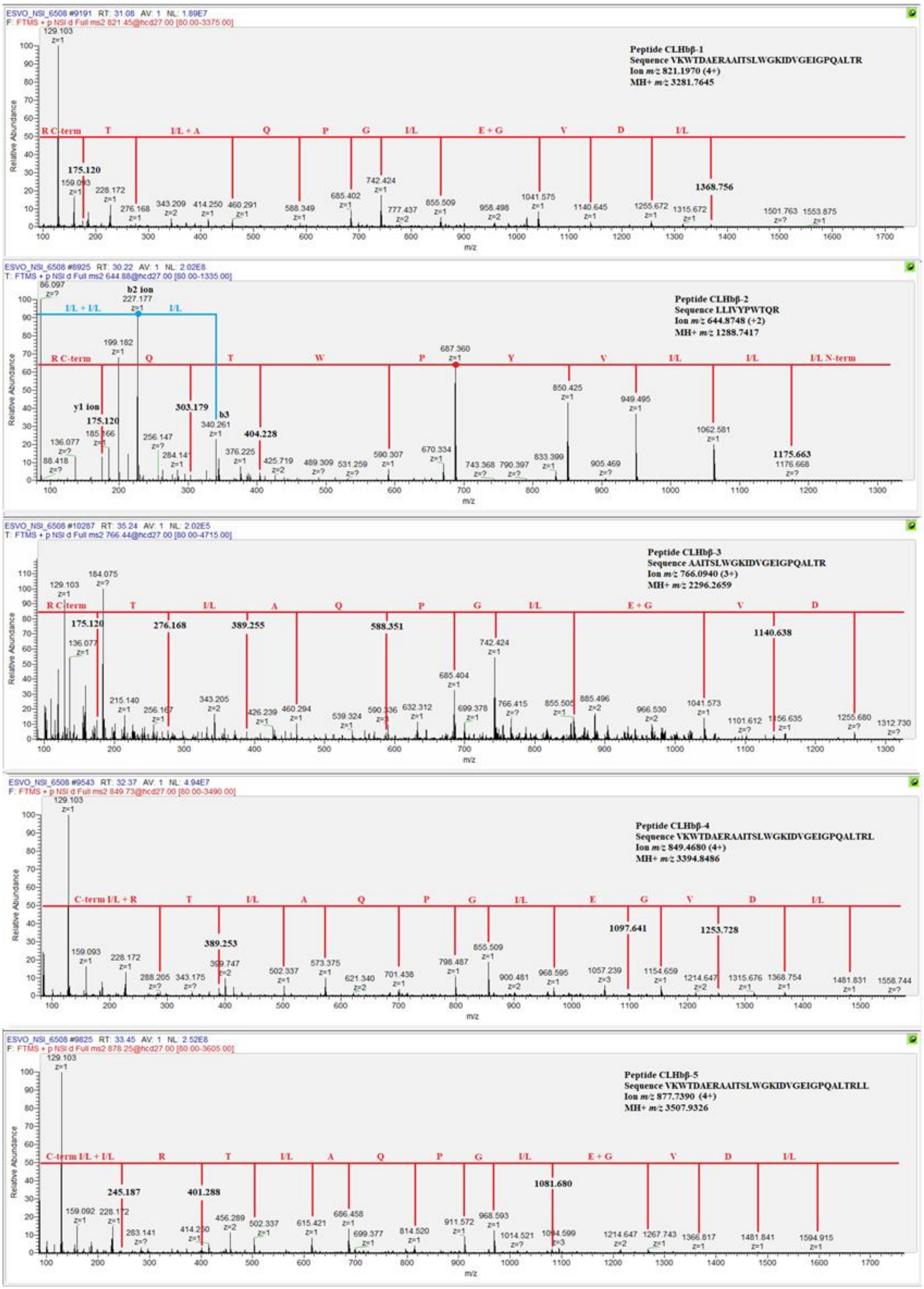

Supplementary Figure 8: MS-MS spectra of the peptides CLHbβ #1 to 5

Peptides isolated from the gill mucus of the butterflyfish *Chaetodon lunulatus* were analysed by nanoLC-MS/MS (see Material-Method for details). We focused on the analysis of five peptides whose presence was revealed by the chromatographic separation. The MS/MS spectra of these peptides were analysed by manual *de novo* sequencing, and the sequence further confirmed by comparison to a protein sequence database (Uniprot). The fragment ions, mostly from the y-ion series, which allowed us to confirm these peptides' sequences have been annotated on these MS/MS spectra. The theoretical *m/z* values for the monoisotopic ion selected for MS/MS and of the MH$^+$ monoisotopic ion are indicated on each spectrum, with the peptide's sequence. C-term, N-term: C-terminal and N-terminal end of the peptide sequence, respectively.
