# Supplementary material for: Insights into the Natural Defenses of a Coral Reef Fish Against Gill Ectoparasites: Integrated Metabolome and Microbiome Approach
Source: Metabolites. 2020 May 30;10(6):227. doi: 10.3390/metabo10060227 (PMC7345202; doi:10.3390/metabo10060227)
Supplement: Supplementary file 1 [file metabolites-10-00227-s001.zip › supplementary/Supplementary material 1.pdf]

Supplementary material 1: Details on the material and method section related to previously published work.

1a: Fish mucus sampling (described in Reverter et al. 2017a and Reverter et al. 2017b)

Fish were spear-fished and killed immediately by brain spiking on the island of Moorea (French Polynesia). Dead fish were put in individual plastic bags with seawater and brought immediately to the laboratory in a cooling box for mucus collection. Gills were aseptically dissected (< 1 hour after spear-fishing them) and mucus was collected by carefully scrapping the gill filaments with a sterile spatula into sterile tubes. Mucus samples for bacterial community analyses were placed on ice until DNA extraction (within the hour following dissection). Mucus samples for metabolomics analyses were freeze-dried and kept at -20°C until chemical extraction.

1b: Untargeted metabolomics pipeline (described in Reverter et al. 2017a)

10 mg of freeze-dried mucus was extracted using a two-step biphasic extraction, yielding two separate fractions. Samples were suspended in 1.5 mL of H_2O/MeOH (2:0.5) and vortexed, and then, 1.5 mL of MeOH/CH_2Cl_2 (2.5:2) was added. The mixture was extracted 15 min in an ultrasonic bath and then centrifuged for 20 min at 3500 x g at 4 °C. The two fractions were carefully collected with glass pipettes into separate tubes. The apolar fraction was dried under nitrogen flux, and the polar fraction was freeze-dried. Polar and apolar fractions were re-suspended in 300 µL MeOH and filtered through a 20-lm polytetrafluoroethylene filter. The quality control (QC) samples were prepared by pooling small aliquots from the polar and apolar extracts.
LC–MS analyses were performed with a LC-DAD-ESI– MS system from ThermoScientific (MA, USA) equipped with an Accela PDA detector and a LCQ Fleet 2300 mass spectrometer with an electrospray ionization source. The equipment was controlled and the data handled by Xcalibur data software. For chromatographic separation, a Kinetex C6-Phenyl analytical column (100 x 2.10 mm, 2.6 µm particle size; Phenomenex, CA, USA) equipped with the corresponding pre-column was used with H_2O (A) and acetonitrile (B) as carriers, both containing 0.1% v/v formic acid. The flow rate was 500 µL min^{-1}, column temperature was maintained at 30 °C and injection volume was 10 µL. For the polar fraction, a linear biphasic gradient from 2 to 100% acetonitrile in water was applied over a period of 30 min, followed by 10-min washing and 15-min re-equilibration with 2% of B before the next injection. For the apolar fraction, a linear gradient was conducted from 30 to 70% of B in 5 min, then from 70 to 80% of B in 20 min and from 80 to 100% of B in 5 min, followed by a 10-min wash with 100% of B and 15-min re-equilibration with 30% of B before the next injection. Full-scan data were acquired in positive ionization mode for both fractions.
The mass spectrometer analyzer parameters were set as follows: sheath gas flow rate 25 a.u. (arbitrary units), aux gas flow rate 5 a.u., sweep gas flow rate 1 a.u., capillary temperature 275 °C, capillary voltage +2 V, tube lens voltage 120 V and spray voltage 4 kV and a full-scan mass window of 130–2000 m/z. To randomize analytical sequences and reduce systematic error associated with instrumental drift, a Latin square was carried out for each of the sample sequences (polar and apolar). QC samples were analyzed at the beginning, the end and equidistantly throughout the sequence. Methanol blank samples were analyzed just before each QC sample to detect column contamination throughout the sequence.
LC–MS raw data files were converted to netcdf files with Excalibur software. Netcdf files were processed using the R package XCMS (R version 3.3.1., XCMS version 1.50.0) to detect, deconvolute and align features (molecular entities with a unique m/z and a specific retention time). Parameter settings for XCMS processing were as follows: matched filter for feature detection (step = 0.5, mzdiff = 0.6, snthresh = 10), peak groups for retention time correction and parameters for chromatogram alignment including mzwid = 0.3, minsamp = 3, bw = 0.5. XCMS analysis of these data provided a matrix containing the retention time, m/z value and integrated peak area of the identified features. Data were normalized by log transformation prior to statistical analysis.

1c: Study of the gill mucus associated bacterial communities (described in Reverter et al. 2017b)

One mL of gill mucus was extracted using the DNeasy Blood & Tissue Kit (Qiagen, Courtaboeuf, France). DNA concentrations were quantified by measuring the absorbance at 260 nm and purity was checked by comparing absorbance at 260:280 nm (> 1.8) and 230:260 nm (> 1.8). DNA samples were sent for 454 pyrosequencing at MRDNALab (Shallower, TX, USA, http://www.mrdnalab.com) using a modified version of a previously published protocol (Croué et al 2013). Briefly, 15 ng DNA was used in 20 μL PCR reactions (94°C, 3 min, followed by 28 cycles of 94°C for 30 s, 53°C for 40 s and 72°C for 1 min; and a final elongation step at 72°C for 5 min) that were performed using the HotStarTaq Plus Master Mix Kit (Qiagen) and primers 27F.1 (5′ AGRGTTTGATCNTGGCTCAG 3′; Kuske et al 2006) and Gray519R (5′ GTNTTACNGCGGCKGCTG 3′; Kostka et al. 2011). The forward primers contained 8-mer tags at the 5′ end to allow multiplexing. The reactions amplified the hypervariable V1–V3 region of the 16S rRNA gene. Products from different samples were mixed in equimolar concentrations and purified using Agencourt Ampure beads (Agencourt Bioscience Co.). Tag-encoded FLX amplicons were sequenced on a Roche 454 FLX sequencer using Titanium (Roche) reagents by MrDnaLab.

Multiplex raw SFF (Standard Flowgram Format) files were analysed using a hybrid analysis pipeline as previously described (Croué et al. 2013) with some modifications. In brief, denoising was done by AmpliconNoise V1.25 (Quince et al. 2011) implemented in Qiime V1.5 (Caporaso et al. 2010) with a small modification to allow it to be run in an iDataplex (IBM) cluster. De novo chimera detection and removal was performed with the uchime module (Edgar 2010; Edgar et al. 2011) of usearch 5.2 (http://drive5.com/usearch/). Non-chimeric sequences were unweighted and grouped into operational taxonomic units (OTUs) with a cut-off of 98% using the usearch v5.2 method implemented in Qiime V1.5. The longest sequence in the OTU was selected as a representative. OTUs were classified using the rdp˙classifier software implemented in Qiime V1.5 (Wang *et al*. 2007) and a database based on the Greengenes August 2013 taxonomy (http://greengenes.secondgenome.com) modified to exclude orders, and corrected to comply with current official nomenclature [(list of prokaryotic names with standing in nomenclature (http://www.bacterio.net)]. Based on this classification, OTUs representing chloroplasts and mitochondria were removed. The resulting OTU table was further treated to remove (using custom bash and awk scripts) OTUs for which the representative sequence was shorter than 372 bp (400 bp minus the length of the primer), OTUs that were represented by a single sequence in the ensemble of samples and a ‘Root’ taxonomy status by the rdp˙classifier analysis, as well as sequences failing aligning by pynast aligner implemented in Qiime. Sequences were aligned and filtered using the Lane mask (Lane 1991) using mothur v1.38.0 (Schloss *et al*. 2009) and a neighbour joining tree necessary to calculate unifrac distances (Lozupone *et al*. 2011) was constructed using phylip v3.6a3 (Felsenstein 1989). A principal component analysis was performed on the preliminary OTU table followed by the calculation of Mahalanobis distances to detect the presence of outliers. One of the samples from *C. vagabundus* was clearly an outlier, and therefore the sample was removed from the OTU table along with all OTUs present exclusively in this sample, as was one OTU abundant in sample CV1 corresponding to a chloroplast, but not identified as such by the RDP classifier.

References:

Caporaso, J.G., Kuczynski, J., Stombaugh, J. *et al.* QIIME allows analysis of high-throughput community sequencing data. *Nat. Methods* **2010**, *7,* 335–336.

Croué, J.,West, N.J., Escande, M-L. *et al.* A single betaproteobacterium dominates the microbial community of the crambescidinecontaining sponge *Crambe crambe*. *Sci. Rep.* **2013**, *3*, 2583.

Edgar, R.C. Search and clustering orders ofmagnitude faster than BLAST. *Bioinformatics* **2010**, *26*, 2460–2461.

Edgar, R.C., Haas, B.J., Clemente, J.C. *et al.* UCHIME improves sensitivity and speed of chimera detection. *Bioinformatics* **2011**, *27*, 2194–2200.

Felsenstein, J. PHYLIP – Phylogeny Inference Package (Version 3.2). *Cladistics* **1989**, *5,* 164–166.

Kostka, J.E., Prakash, O., Overholt, W.A. *et al.* Hydrocarbon-degrading bacteria and the bacterial community response in Gulf of Mexico beach sands impacted by the deepwater horizon oil spill. *Appl. Environ. Microbio.l* **2011**, *77*, 7962–7974.

Kuske, C.R., Barns, S.M., Grow, C.C. *et al.* Environmental survey for four pathogenic bacteria and closely related species using phylogenetic and functional genes. *J. Forens. Sci.* **2006**, *51*, 548–558.

Quince, C., Lanzen, A., Davenport, R.J. *et al.* Removing noise from pyrosequenced amplicons. *BMC Bioinformatics* **2011**, *12*, 38.

Reverter, M.; Sasal, P.; Banaigs, B.; Lecchini, D.; Lecellier, G.; Tapissier-Bontemps, N. Fish mucus metabolome reveals fish life-history traits. Coral Reefs 2017a, 36, 463–475, doi:10.1007/s00338-017-1554-0.

Reverter, M.; Sasal, P.; Tapissier-Bontemps, N.; Lecchini, D.; Suzuki, M. Characterisation of the gill mucosal bacterial communities of four butterflyfish species: a reservoir of bacterial diversity in coral reef ecosystems. FEMS Microbiol. Ecol. 2017b, 93, 051, doi:10.1093/femsec/fix051.

Schloss, P.D., Westcott, S.L., Ryabin, T. *et al.* Introducing mothur: open-source, platform-independent, community-supported software for describing and comparing microbial communities. *Appl. Environ. Microbiol.* **2009**, *75*, 7537–7541.

Wang, Q., Garrity, G.M., Tiedje, J.M. *et al.* Naıve Bayesian classifier for rapid assignment of rRNA sequences into the new bacterial taxonomy. *Appl. Environ. Microbiol.* **2007**, *73*, 5261–5267.
